# A simple method to describe the COVID-19 trajectory and dynamics in any country based on Johnson cumulative density function fitting

**DOI:** 10.1038/s41598-021-97285-5

**Published:** 2021-09-07

**Authors:** Adam M. Ćmiel, Bogdan Ćmiel

**Affiliations:** 1 Institute of Nature Conservation, Polish Academy of Sciences, al. A. Mickiewicza 33, 31-120 Kraków, Poland; 2 Faculty of Applied Mathematics, AGH University of Science and Technology, al. A. Mickiewicza 30, 30-059 Kraków, Poland

**Keywords:** Diseases, Biological techniques

## Abstract

A simple method is utilised to study and compare COVID-19 infection dynamics between countries based on curve fitting to publicly shared data of confirmed COVID-19 infections. The method was tested using data from 80 countries from 6 continents. We found that Johnson cumulative density functions (CDFs) were extremely well fitted to the data (R^2^ > 0.99) and that Johnson CDFs were much better fitted to the tails of the data than either the commonly used normal or lognormal CDFs. Fitted Johnson CDFs can be used to obtain basic parameters of the infection wave, such as the percentage of the population infected during an infection wave, the days of the start, peak and end of the infection wave, and the duration of the wave’s increase and decrease. These parameters can be easily interpreted biologically and used both for describing infection wave dynamics and in further statistical analysis. The usefulness of the parameters obtained was analysed with respect to the relation between the gross domestic product (GDP) per capita, the population density, the percentage of the population infected during an infection wave, the starting day and the duration of the infection wave in the 80 countries. We found that all the above parameters were significantly associated with GDP per capita, but only the percentage of the population infected was significantly associated with population density. If used with caution, this method has a limited ability to predict the future trajectory and parameters of an ongoing infection wave.

## Introduction

A highly contagious disease, COVID-19, is caused by the SARS-CoV-2 coronavirus. This virus was first detected in Wuhan (central China) in December 2019, but as early as mid-January 2020, the virus had quickly spread throughout China. On 13 January 2020, the first case outside China was confirmed, and on 24 January, the first case in Europe was reported. By the second half of February 2020, outbreaks with hundreds of cases had erupted in South Korea, Italy and Iran^1^, and on 11 March 2020, COVID-19 was declared a pandemic by the World Health Organization^2^. To date, over 180 million infections and almost 4 million deaths have been reported globally^3^.

Since the very beginning of the pandemic, many models have been proposed to understand the outbreak dynamics of COVID-19^4–9^ and have been used by policymakers (e.g., the US government) for allocating resources or planning interventions. Some of them, such as the early IHME model, have received a fair amount of criticism^10^. COVID-19 modelling studies generally follow one of two general approaches—forecasting models and mechanistic models—although there are also hybrid approaches^11^. Forecasting models are often statistical in nature, fitting a line or a curve to data and extrapolating from there, without incorporating the process that produces the pattern^11^, while mechanistic models simulate the outbreak through interacting disease mechanisms by using local nonlinear population dynamics and the global mixing of populations^12^. Purely statistical models are reliable only within a short time window and may be useful for making rapid, short-term recommendations, whereas mechanistic modelling can be useful for exploring how the course of the pandemic might change under various assumptions and political interventions^13^.

Since its onset, the COVID-19 pandemic has generated a huge amount of data and is probably the best documented disease in history. New cases, active cases, deaths and the number of tests performed are usually published daily by official sources (e.g., governments), gathered and publicly shared as freely accessible datasets (e.g.,^14^). This offers researchers an opportunity to focus on analysing the pandemic and its dynamics in fields other than epidemiology. Although the abovementioned models provide many pandemic parameters for predicting different scenarios of future infections, the probability and duration of future pandemic peaks, which is extremely useful for policymakers in planning interventions, may not be very useful in fields other than epidemiology. Thus, there is an urgent need to develop methods to describe the trajectories of pandemic waves. Such methods should be simple to apply and should provide parameters explaining the trajectory and dynamics of the epidemic that are easy to interpret and employ in further statistical analyses by researchers in other fields, such as sociology, biology and ecology, which can deepen our understanding of the COVID-19 pandemic.

Curve fitting methods are a valuable tool to understand epidemic data that are not sufficiently used in practice and thus worth popularising. The aim of this paper is to present a simple method based on curve fitting to the reported data on confirmed cases of infection and to study and compare the infection dynamics between countries (or regions). This method, based on Johnson cumulative density function (CDF) fitting to the cumulative epidemic curves, was tested using data from 80 countries from 6 continents (Africa, Asia, Europe, Oceania, and North and South America). Additionally, Johnson CDFs were used to calculate basic parameters of the infection wave dynamics, such as the percentage of the population infected during the infection wave, the days of the start, peak and end of the infection wave, the duration of the infection wave, and the duration of the wave’s increase and decrease. These parameters are simple to interpret and can be used in further statistical analyses of epidemic dynamics. This is exemplified by the indirect influences of gross domestic product (GDP) per capita and population density on the percentage of infections and the first day and duration of the first infection wave in the countries concerned. The method presented and the techniques employed are both straightforward and well known; they illustrate how simple techniques can be used to solve otherwise complex problems, such as describing an epidemic wave.

## Materials and methods

The data used in this study were obtained from the Our World in Data COVID-19 dataset^14^ from 30 December 2019 to 19 October 2020. The method was tested on 80 countries from 6 regions: (1) Africa (Democratic Republic of Congo, Egypt, Ethiopia, Kenya, Morocco, Nigeria, Somalia, South Africa, South Sudan, Sudan and Zimbabwe); (2) Asia (Afghanistan, Bangladesh, Cambodia, China, India, Indonesia, Iran, Iraq, Israel, Japan, Lebanon, Myanmar, Pakistan, Philippines, Saudi Arabia, Singapore, South Korea, Sri Lanka, Syria, Taiwan, Thailand, Turkey and Vietnam); (3) Europe (Austria, Belgium, Bosnia and Herzegovina, Bulgaria, Croatia, Cyprus, Czechia, Finland, France, Germany, Greece, Hungary, Ireland, Italy, Netherlands, North Macedonia, Norway, Poland, Portugal, Romania, Russia, Serbia, Slovakia, Slovenia, Spain, Sweden, Switzerland, Ukraine and the United Kingdom); (4) North America (Canada, Jamaica, Mexico and the United States of America); (5) Oceania (Australia, Fiji, New Zealand and Papua New Guinea); and (6) South America (Argentina, Bolivia, Brazil, Chile, Colombia, Paraguay, Peru, Uruguay and Venezuela).

To make the data comparable between countries, the cumulative number of infections on each day of the pandemic for each country was standardised; they are presented here as a cumulative percentage of the population of a given country infected, i.e., the cumulative number of confirmed infections in a given country/country population * 100%. Additionally, a 5-day moving average was calculated using the cumulative percentage of infections to smooth the data. This makes the loss function more regular, i.e., it has fewer relative extrema, so it is easier to find the global extremum and decreases the sensitivity of the numerical estimation method to changes in the starting point values (more detail in the Supplementary Materials). Nevertheless, all the presented coefficients of determination (fraction of the explained variance; R^2^) for the Johnson CDFs obtained were calculated using raw (not smoothed) data.

### Fitting Johnson unbounded (Johnson S_U_) CDF by moments

Johnson^15^ described a system of frequency curves that represent transformations of the standard normal curve. Applying these transformations allows a unique curve to be derived for whatever combination of mean, standard deviation, skewness and kurtosis occurs for a given set of observed data. The standard method of fitting Johnson curves is to use four coefficients defining a Johnson distribution: two shape coefficients (*γ*, *δ*) as well as a location (*ξ*) and a scale (*λ*) coefficient:

1 $$F\left(x\right)=\Phi \left(\gamma +\delta {sinh}^{-1}\left(\frac{x-\upxi }{\lambda }\right)\right),$$

where Φ is the standard normal CDF.

This method is not intuitive, however, as it is difficult to set up starting points from the data to perform numerical fitting. Thus, an alternative method for fitting Johnson curves using the first four moments (mean, standard deviation, skewness and kurtosis; detailed descriptions in^16,17^) was selected. All the statistical fits in the paper were performed using the Levenberg–Marquardt algorithm^18^ to solve the corresponding nonlinear least square optimisation problem. The convergence criterion was set at 1.0E^−10^, while the maximum number of iterations was set at 10,000.

### Fitting Johnson CDFs to epidemic waves

There is no strict definition of what is or is not an epidemic wave or phase. The intuitive definition of a pandemic wave traces the development of an epidemic over time and/or space. During an epidemic, the number of new cases of infection increases (often rapidly) to a peak and then falls (usually more gradually) until the epidemic wave is over. Each epidemic wave may be visualised by an epidemic curve. To visualise an epidemic curve, we put the number of cases on the vertical axis and the time unit on the horizontal axis^19^. Another possible way of visualising an epidemic wave is to place the cumulative number of cases on the vertical axis. In such cases, we obtain a cumulative epidemic curve (sigmoid shape instead of a "wave-like" shape). Nevertheless, the cumulative epidemic curve, even if it does not present the wave shape itself, describes the same epidemic wave or phase as the epidemic curve. Epidemic dynamics may differ greatly between countries. Since the beginning of the pandemic, there has been only one epidemic wave in some countries (e.g., Afghanistan, Argentina), while in others, two have occurred (e.g., Australia); in yet others, even more have taken place, which may have overlapped and interfered with each other, as in Croatia, where there were four overlapping and interfering waves. Moreover, authorities in many countries have imposed lockdowns of varying degrees of severity to slow down or "flatten" the infection curve. Hence, epidemic waves may not follow Farr's law (which states that epidemics tend to rise and fall in a roughly symmetrical pattern or bell-shaped curve) and may be asymmetrical.

The basic assumption is that each epidemic wave *W* in a given country, visualised by a cumulative epidemic curve (cumulative number of infections in time), can be described by a five-parameter scaled Johnson unbounded (S_U_) CDF: scale parameter (*s*), and the abovementioned moments–expected value (mean, *E*), standard deviation (*σ*), skewness (*S*) and kurtosis (*K*),

2 $$W\left( t \right) = s*F_{E,\sigma ,S,K} (t),$$

where *t* is the time measured since the day of the beginning of the pandemic, and the function *F*_*E,σ,S,K*_ is the Johnson CDF with parameters *γ*, *δ, ξ,* and *λ*, assuming the mean, standard deviation, skewness and kurtosis to be equal to *E,σ,S,* and *K*, respectively. Parameters *S* and *K* were expected to improve the curve fit at the tails of the epidemic wave if it was not symmetrical or heavy tailed. A more detailed description and examples of Johnson CDF fitting to the cumulative epidemic curve are presented in the Supplementary Materials (pp. 2–9; Tables S1–S3; Figs. S1–S4).

### Obtaining basic epidemic wave parameters and their biological interpretation

Once the Johnson CDFs were fitted to each pandemic wave in a given country, the basic parameters for obtaining the wave dynamics, i.e., 2.5% quantile (*Q*_*2.5%*_), 50% quantile (median; *Q*_*50%*_), 97.5% quantile (*Q*_*97.5%*_), could be calculated:

3 $${Q}_{2.5\%}={F}_{E,\sigma ,S,K}^{-1}(2.5\%)$$

4 $${Q}_{50\%}={F}_{E,\sigma ,S,K}^{-1}(50\%)$$

5 $${Q}_{97.5\%}={F}_{E,\sigma ,S,K}^{-1}(97.5\%)$$

The disadvantage of fitting the Johnson curve by its moments is that it is not possible to determine its mode analytically. Thus, the mode of each Johnson CDF was determined numerically:

6 $$M=\mathrm{argmax}{f}_{E,\sigma ,S,K}\left(x\right),$$

where ${f}_{E,\sigma ,S,K}$ is the Johnson density function

7 $${f}_{E,\sigma ,S,K}\left(t\right)=\frac{d}{dt}F_{E,\sigma ,S,K} \left(t\right).$$

**Figure 1:**
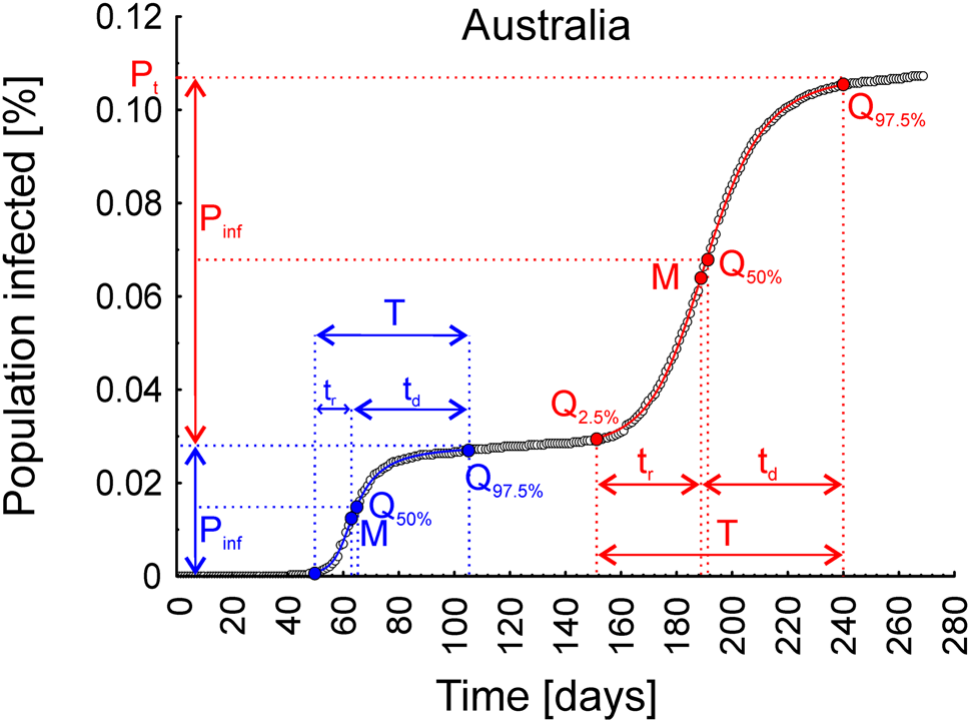
Graphical presentation of the interpretation of the parameters obtained from the Johnson cumulative density function fitted to the cumulative epidemic curve, describing the dynamics of the two infection waves observed in Australia. *P*_*inf*_—the total percentage of infections in a given infection wave, *Q*_*2.5%*_—the day the infection wave started, *Q*_*97.5%*_—the day the infection wave ended, *Q*_*50%*_—the day that half of the total percentage of infections during a given wave was reached, *M*—the day the infection wave peaked, *T*—the duration of the wave, *t*_*i*_—the duration of the wave increase, *t*_*d*_—the duration of the wave decrease, and*P*_*t*_—the total percentage of the population infected after two infection waves.

These parameters have an intuitive biological interpretation (Fig. 1). The scale parameter *s* indicates the total percentage of infections during a given epidemic wave (*P*_*inf*_), *Q*_*2.5%*_ indicates the day when the infection wave started, and *Q*_*97.5%*_ indicates its end. The median (*Q*_*50%*_) indicates the day when half the total percentage of infections during a given wave was reached. Finally, mode (*M*) indicates the day when the peak occurred. In addition, one can easily obtain the wave duration (*T*)

8 $$T = Q_{97.5\% } - Q_{2.5\% } ,$$

the duration of wave increase (*t*_*i*_)

9 $$t_{i} = M \, - \, Q_{2.5\% } ,$$

and the duration of the wave decrease (*t*_*d*_)

10 $$t_{d} = Q_{97.5\% } - \, M.$$

Additionally, the parameter measuring the asymmetry of infection wave (*A*) is easily obtained as the ratio

11 $$A = t_{i} /t_{d} .$$

All the above mentioned parameters can be easily used in further statistical analyses, as exemplified by (1) the statistical dependence between GDP per capita and the basic parameters describing the dynamics of the first wave of infections (*M*, *T* and *P*_*inf*_) and (2) the relation between population density and the basic parameters describing the dynamics of the first wave of infections (*M*, *T* and *P*_*inf*_). Only the first wave of infections in each country has been taken into account here because in some countries, second (and subsequent) waves did not occur and would have to be excluded from the analysis. The basic parameters of the first infection wave dynamics (*S*, *P*_*inf*_, *Q*_*2.5%*_, *Q*_*50%*_, *Q*_*97.5%*_, *M*, *t*_*i*_, *t*_*d*_, *T*, *A*) calculated using Johnson CDFs fitted to the data obtained for 80 countries on six continents are listed in the Supplementary Materials (Table S9, pp. 37–39).

### Sensitivity analysis

Sensitivity analysis was performed to check (1) the sensitivity of the numerical algorithm to data perturbation, (2) the sensitivity of the algorithm to changes in selected starting point values, (3) the sensitivity of the fitted curve to the change in its parameter values and (4) the influence of smoothing the raw data on the sensitivity of the algorithm to changes in selected starting point values. A more detailed description and results of the sensitivity analysis are presented in the Supplementary Materials (pp. 10–15; Tables S4–S7; Fig. S5).

### Comparing curves: Johnson vs normal and lognormal CDFs

The differences between the Johnson, normal and lognormal CDFs were highlighted on the basis of data from Afghanistan, where only one epidemic wave took place, by comparing parameters R^2^, *P*_*inf*_, *Q*_*2.5%*_, *M* and *Q*_*97.5%*_. Both the 2.5% and 97.5% quantiles for normal and lognormal curves were obtained using inverse normal and inverse lognormal density functions, respectively.

### Fitting Johnson’s CDF to the ongoing wave and forecast possibilities

Fitting Johnson’s curve to the ongoing wave yields parameters that can also be interpreted as a forecast of the future shape and dynamics of the infection wave. In such a case, *P*_*inf*_, *M* and *Q*_*97.5%*_ indicate the predicted percentage of infections, the predicted day of the peak and the predicted day of the end of the ongoing wave, respectively, which can also be used to calculate the predicted times of the increase, decrease and duration of the ongoing infection wave. Because this method is intended to describe the infection dynamics rather than to predict its ultimate outcome, the accuracy of the forecast is evaluated only on the basis of data from the first wave of infection recorded in the United Kingdom (see Supplementary Materials, pp. 40–42).

### Examples of application

#### The statistical dependence between gross domestic product (GDP) per capita and population density and the dynamics of the first wave of COVID-19 infections

The data on the GDP per capita and population density in the 80 countries analysed here were obtained from the Our World in Data COVID-19 dataset^14^.

The statistical dependence between GDP per capita and population density and the basic parameters describing the dynamics of the first wave of infections (*M*, *T* and *P*_*inf*_), obtained using the presented method of Johnson CDF fitting, was tested using the quantile dependence function method, described in detail in^20^. This method was designed for measuring, visualising the dependence structure, and testing the independence of two random variables. It exploits a recently introduced local dependence measure (quantile dependence function *q*), which gives a detailed picture of the underlying dependence structure and provides a means by which the local association structure can be minutely examined at different quantile levels^20^.

## Results

**Figure 2:**
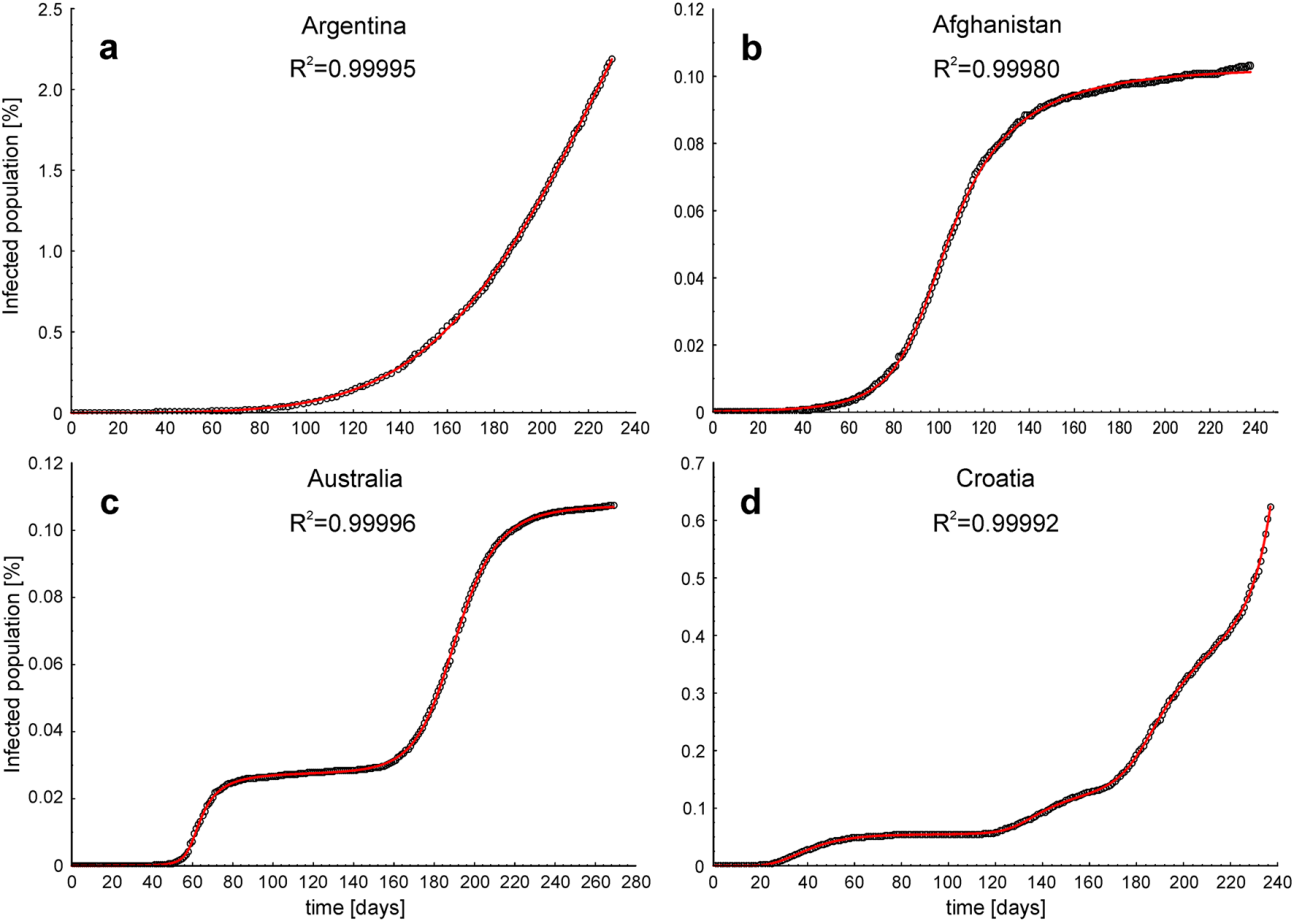
Examples of Johnson cumulative density functions fitted to the cumulative epidemic curves in four scenarios of COVID-19 infection dynamics. (**a**) One ongoing infection wave (before the peak), (**b**) one full wave, (**c**) two waves and (**d**) four overlapping and interfering waves. Open circles—raw data, red lines—fitted Johnson CDFs.

**Figure 3:**
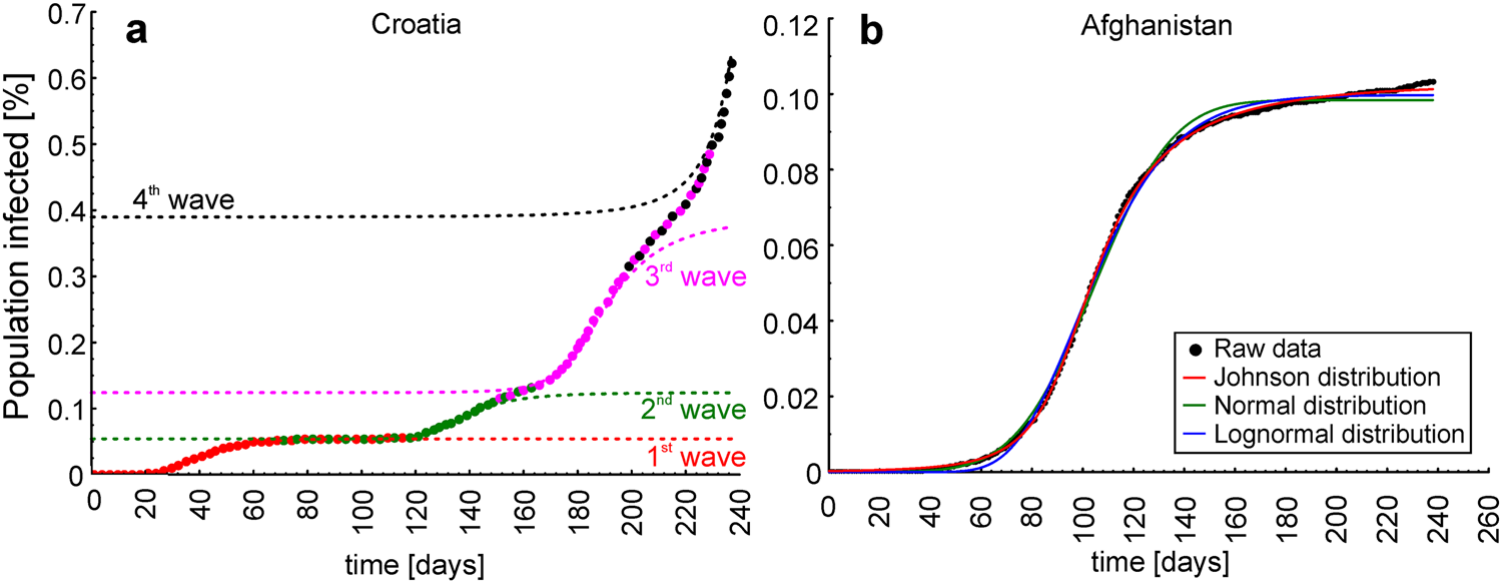
(**a**) Trajectory of four Johnson cumulative density functions fitted to the cumulative epidemic curve reported in Croatia, showing four waves of infections with the areas where the waves overlapped and interfered, and (**b**) the differences between Johnson (red line), normal (green line) and lognormal (blue line) cumulative density functions fitted to the cumulative epidemic curve from Afghanistan (black dots).

Figure 2 illustrates examples of Johnson CDFs fitted to the cumulative epidemic curves from countries where one ongoing infection wave (Argentina), one infection wave (Afghanistan), two infection waves (Australia) and four overlapping and interfering infection waves (Croatia) occurred. Figure 3a shows Johnson CDFs fitted to the cumulative epidemic curve reported in Croatia, showing four waves of infections with the areas where the waves overlapped and interfered.

Johnson CDF fitting, tested using data from 80 different countries, showed that all the curves were extremely well fitted: the lowest R^2^ was 0.995 (Fiji), the highest R^2^ was 0.99997 (Iraq), and the mean and median R^2^ were 0.9995 and 0.9997, respectively. The functions fitted with R^2^ and the COVID-19 trajectory plots with fitted functions for each country are illustrated in the Supplementary Materials (pp. 16–38; Table S8; Figs. S6–S11).

The sensitivity analysis results showed that the fitted Johnson CDF was very sensitive to data perturbation and errors, but only when it was fitted using just 30% of the available data (72 days of the ongoing infection wave, far before the cumulative epidemic curve inflexion point). When 40% of the available data (96 days of the ongoing infection wave, just before the cumulative epidemic curve inflexion point) was used to fit the Johnson CDF, its sensitivity to data perturbation greatly decreased, while it was hardly sensitive to data perturbation when 50% of the data (120 days of the ongoing wave, after the inflexion point of the cumulative epidemic curve) was used in the estimation (Supplementary Materials; Table S4; Fig. S5). Moreover, sensitivity analysis revealed that the numerical algorithm used was hardly sensitive to the changes in values of selected starting points, providing stable parameter estimates (Supplementary Materials; Table S5) and that the fitted Johnson CDF was the most sensitive to the changes in the values of the *s* and *E* parameters. Changing the value of these parameters by more than ± 5% resulted in a relatively high decrease in the R^2^ value, whereas for other parameters (*σ*, *S*, *K*), the R^2^ value was still higher than 0.99, even after changing the value of the parameters by ± 25% (Supplementary Materials; Table S6). Finally, sensitivity analysis showed that when fitting Johnson CDF to the ongoing wave, smoothing the raw data makes the numerical algorithm less sensitive to the changes in the values of the selected starting points (Supplementary Materials; Table S7).

Fitting Johnson, normal and lognormal curves to the single wave of infections that took place in Afghanistan showed that the Johnson CDF (R^2^ = 0.9998) was the best fitted, whereas the normal (R^2^ = 0.9980) and lognormal (R^2^ = 0.9989) curves were not as well fitted, especially at the tails of the infection wave (Fig. 3b). The parameters *Q*_*2.5%*_, *M* and *Q*_*97.5%*_ obtained for the infection wave in Afghanistan using the Johnson CDF fitting were 59, 100 and 209, respectively, whereas the same parameters obtained using the normal CDF fitting and lognormal CDF fitting were 57, 105 and 152 and 65, 98 and 167, respectively. The percentages of the population infected during the infection wave obtained using the scale parameters (*s*) of the fitted Johnson, normal and lognormal curves were 0.1028%, 0.0984% and 0.0997%, respectively.

Seventeen (21.3%) of the 80 countries analysed were described by fitting one infection wave, while 35 (43.8%), 24 (30%) and 4 (5%) were described by fitting two, three and four infection waves, respectively (Table S8).

The basic statistics for the skewness parameters of the Johnson CDFs fitted to the first pandemic waves in the 80 counties showed that the majority of the first infection waves were skewed (median *S* = 1.5; minimum *S* = 0; maximum *S* = 141.5; Table S2). The first wave of infections was symmetrical in 16 countries (20%; *A* < 1.05). Additionally, the basic statistics for parameter *A* showed that the duration of the wave decrease was longer than that of the wave increase (mean *A* = 4.7; median *A* = 2.9; minimum *A* = 1.0; maximum *A* = 22.4; Table S9).

**Figure 4:**
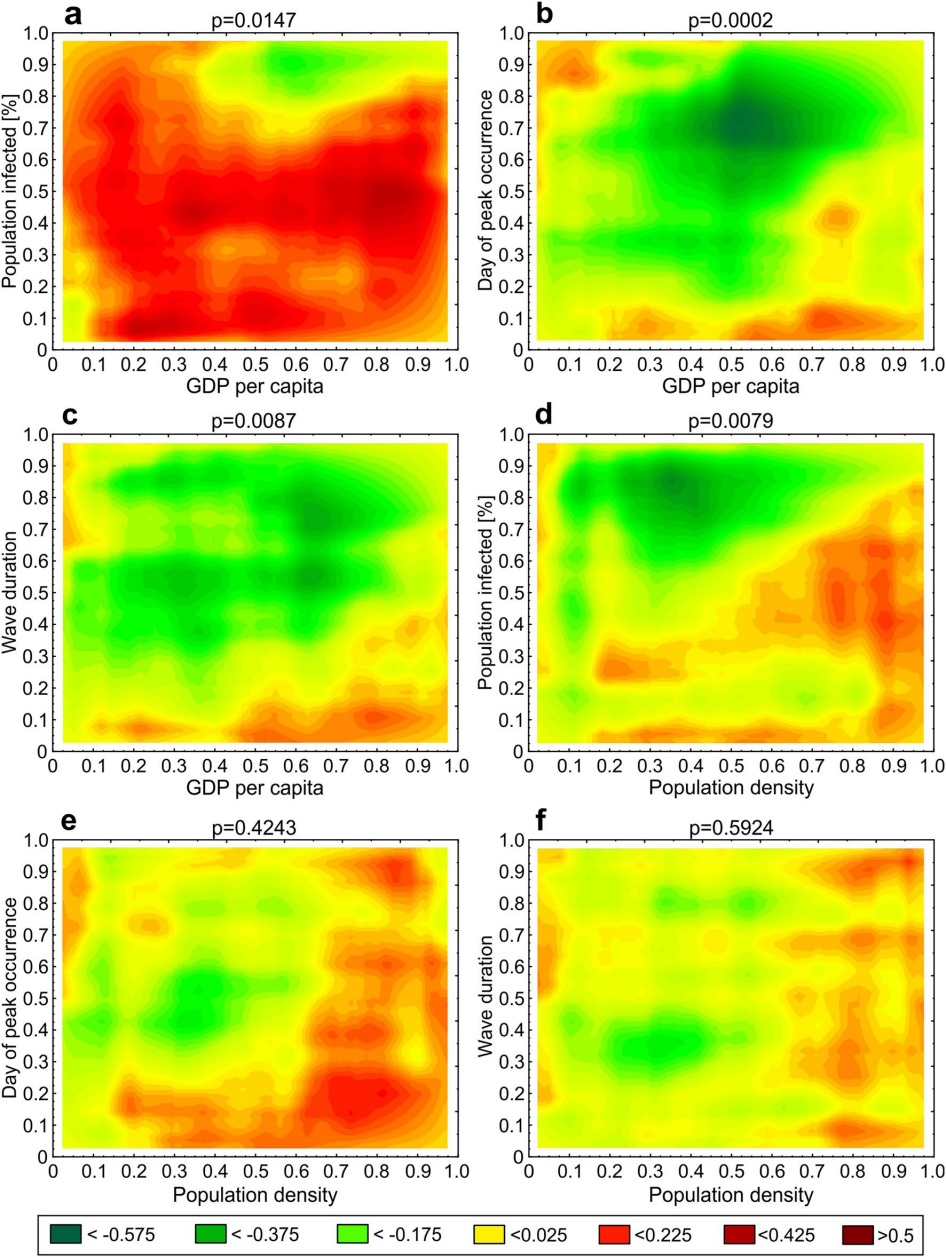
Heat maps showing *p* values and the local association structure between variables at different quantile levels obtained using the quantile dependence function *q.*

An analysis of the associations between GDP per capita and parameters *M*, *T* and *P*_*inf*_ showed that the percentage of confirmed infections during the first epidemic wave in the 80 countries was significantly associated with the GDP per capita (p = 0.0147; Fig. 4a), the time of the peak (*M*; p = 0.0002; Fig. 4b) and the duration of the first epidemic wave (*T*; p = 0.0087; Fig. 4c). The association of the percentage of infections with GDP per capita tended towards a global positive dependence (Fig. 4a), meaning that the higher the GDP per capita, the greater the percentage of infections during the first epidemic wave. The association of the time of the peak with GDP per capita showed a local negative dependence for countries where the peak occurred late (above median; Fig. 4b). This means that the very early occurrence of a peak was not associated with GDP per capita; however, in those cases when the peak did not occur early, the higher the GDP per capita, the earlier the peak occurred. A similar situation prevailed for the association between the duration of the infection wave and GDP per capita (Fig. 4c), i.e., a very short first epidemic wave was not associated with GDP per capita. In contrast, in those cases where the first epidemic wave was of a longer duration, the higher the GDP per capita, the shorter the first wave.

An analysis of the associations between population density and parameters *M*, *T* and *P*_*inf*_ showed that the percentage of infections during the first epidemic wave in the 80 countries was significantly associated with population density (p = 0.0079; Fig. 4d), whereas the day of the peak and the duration of the first epidemic wave were not (*T*: p = 0.4243; Fig. 4e; *M*: p = 0.5924; Fig. 4f). The association of the percentage of infections with population density showed a local negative dependence (Fig. 4d), i.e., in those cases where the population density was low, the percentage of infections was quite high, and conversely, the percentage of infections was low in cases with a high population density.

## Discussion

The method presented in this paper provides information about the dynamic of the spread of COVID-19 in any particular country that publicises daily numbers of infected cases. Both this method and the techniques employed are straightforward, well known and easy to use, since the Johnson CDF fitting is available in many statistical/calculus packages, e.g., R, Statistica, MATLAB, and MS Excel. By using an alternative method of fitting that uses moments instead of shape, location and scale parameters, it is easier to set starting points for the numerical fitting, e.g., by visually analysing the scatter plot of the number of infected cases in time. The curves are extremely well fitted; this is exemplified by the data from 80 different countries on 6 continents. Additionally, the parameters are easy to interpret and ready to use in further analyses, such as finding associations between them and other variables that may be associated with COVID-19 dynamics, such as GDP per capita and population density.

Curve fitting methods are a valuable tool to understand epidemic data. Some researchers used the Richards integral curve to describe cumulative reported case data of the 2003 SARS outbreaks in Beijing, Hong Kong, Singapore^21^, and Taiwan^22^, as well as to fit COVID-19 data and recover epidemic parameters from it^23^; the parameters of the Richards curve in connection with the SIR model were further discussed by Wang, Wu and Yang^24^. Additionally, some researchers have used curve fitting with a normal distribution to respond to a real-time request, applying it to COVID-19 in Wuhan^25^, since it was known that flu epidemics follow a normal distribution, whereas other researchers noticed that the COVID-19 profile had a characteristically asymmetric tail and applied lognormal distribution curve fitting^26^. However, fitting normal and lognormal distributions to the epidemic curves is confusing because, although an epidemic curve may look like a Gaussian curve and be eventually modelled by a Gaussian function, it is not a normal or a lognormal distribution (see^19^ for details). Moreover, our results show that the first wave of infections was highly skewed in 79% of the countries analysed. This suggests that, unlike flu epidemics, the COVID-19 epidemic does not follow a normal density function and should not be modelled in this manner. In such a case, log-normal density function fitting appears to be better suited; however, as the example of Afghanistan shows, the apparent differences in R^2^ between the Johnson, normal and lognormal CDFs are small but are in fact approximately one order of magnitude in favour of the Johnson CDF. Moreover, one can see that both the normal and lognormal CDFs are not as well fitted at the tails of the infection wave as the Johnson CDF (Fig. 3b). Additionally, both reveal a smaller number of infections than were actually recorded (raw data) and fewer than what were obtained using Johnson’s CDF. The fitted lognormal curve also starts to increase later than the normal and Johnson distribution curves, which would, in consequence, lead to an incorrect estimate of the beginning of the wave (11 days later than when obtained using Johnson’s CDF), whereas the normal curve fits far more poorly at the right tail than the Johnson and lognormal curves because the infection wave in Afghanistan was not symmetrical. Although using the normal curve would preclude any estimate of the true duration of the wave decrease (by definition, equal to the time of the wave increase), it also leads to a much lower estimate of the day when the wave of infections ends (57 days earlier than estimated using the Johnson distribution), which is caused by the "too fast" flattening of the normal CDF (Fig. 3b). The extremely high R^2^ values obtained for the 80 countries (see Supplementary Materials, Table S8) suggest that the Johnson curve class is flexible enough to almost perfectly follow the course of the epidemic in these countries. This is because both skewness and kurtosis are estimated during the Johnson curve-fitting procedure, whereas the shapes of other commonly used curves (normal, lognormal, Weibull) are more or less imposed. This result also suggests that the Johnson CDF should be the preferred curve-fitting approach for COVID-19 data.

The curve-fitting method presented here was designed primarily to obtain easily interpretable parameters describing past trajectories of COVID-19 infections, but the parameters describing the current wave of infections, especially in its early stages (before the peak), can be interpreted as a forecast of the future course of the pandemic. In such a case, however, extreme caution is advisable (see^10^). This method is a purely statistical model that does not incorporate the process that produces the pattern of the number of infections and does not account for parameters governing transmission, disease and immunity. In addition, curve-fitting techniques cannot predict the occurrence of future peaks. Moreover, sensitivity analysis showed that fitting Johnson CDFs to the ongoing wave of infections, especially at the beginning of the infection wave when the inflexion point of the cumulative epidemic curve is still not visible, is sensitive to data perturbations. Thus, the uncertainty of such estimates is high and can be used only as a rough indicator with very low reliability. Thus, for long-term forecasts and future modelling scenarios of the pandemic, it is recommended that more reliable methods be used, such as those based on susceptible-exposed-infectious-recovered (SEIR) models. Nevertheless, when fitting Johnson CDFs to the ongoing wave of infections with visible inflexion points, the estimates are much more reliable; in this case, some short-term predictions can be made, which may be useful to policymakers for planning rapid, short-term interventions. One must bear in mind, however, the abovementioned method’s limitations, as well as those resulting from the data collection and reporting, which are discussed later in this section. Final conclusions should also be supported by intensive care internments, the mortality rate, and the imposed policies of lockdown; otherwise, conclusions maybe incomplete.

The results obtained from this application of parameters describing COVID-19 dynamics have shown that the higher the GDP per capita is, the higher the percentage of the population infected. This is quite an unexpected result but is consistent with a recent report by Liu et al.^27^, who found a positive correlation between the human development index (HDI) and the risk of infection and death from COVID-19 in Italy. Other results have shown that, excluding countries where the infection wave peaked very early and was of a short duration, the higher the GDP per capita, the earlier the peak and the shorter the first epidemic wave. This result, in turn, is similar to that reported in another recent paper, in which the date of the first COVID-19 cases was shown to covary positively with GDP across countries, most likely because of closer involvement of these countries in global tourism and transport^28^. However, the significant positive association of GDP per capita with the percentage of the population infected may be a result of differences in the capacity to detect infections between high- and low-GPD countries. Since most COVID-19 cases are asymptomatic**,** countries with lower GDP may not be equipped to detect them, in which case the trace of the infection wave may be affected and underestimated. Moreover, the testing procedure involves a substantial number of techniques and skills, from medical doctors to nurses and laboratory technicians. The availability of people in the population who possess those skills is probably correlated with the higher education system and health system of the country, which in turn is also correlated with GDP. Therefore, there might be a greater underreporting of cases in low-GDP countries than in high-GDP countries. Additionally, one should keep in mind that the relationship between GDP and epidemic parameters may be more complex than the quantile dependence function can show and that until more infection waves are analysed, the associations will not be robust since it is already known that the dynamics of different waves are related to other natural parameters (e.g., susceptibility, age, meteorological and environmental influences).

Another example showed that the greater the population density, the lower the percentage of the population infected during the first wave of infections. Although this too seems unexpected, the negative dependence may result from the fact that infections are presented as a percentage, which does not scale proportionally with population density. A further possible explanation is that in countries with a high population density, such as China and Singapore, very strict (full) lockdowns were immediately applied (China^29^; Singapore^30^), which may have resulted in fewer people being infected than in countries with a lower population density, where lockdowns were only partial, if imposed at all. Moreover, some researchers report a positive correlation between population density and the number of infections and related mortality, e.g., in India^31^, whereas others provide no evidence that population density is linked with COVID-19 cases and deaths, e.g., in the US^32^. Nevertheless, these examples demonstrate the usefulness of our method. Recent papers by^27,28^ have also shown that the volume of research on COVID-19, other than purely epidemiological modelling of future pandemic scenarios, is increasing. This indicates that simple methods of obtaining parameters describing infection waves, such as those presented in this paper, may be very useful can help deepen our understanding of the COVID-19 pandemic.

Last but not least is the issue that our understanding of the COVID-19 pandemic is limited in that the true number of infections is unknown and the only infections that are known are those confirmed by tests. Moreover, testing strategies differ between countries; in some countries, only symptomatic cases are tested, while in others, mass testing is carried out. Moreover, most COVID-19 cases are asymptomatic and remain unreported^33^. Consequently, mortality data are generally considered more reliable than the testing-dependent confirmed case counts that are used in COVID-19 epidemic modelling (e.g.^34^). However, some countries only report COVID-19 deaths that occur in hospitals, whereas others report COVID-19 deaths when a test has confirmed the infection (this makes the number of mortalities testing-dependent as well). On the other hand, if a laboratory diagnosis is not required, as in the UK^35^, it is possible that deaths due to other diseases with COVID-19-like symptoms are reported as COVID-19 deaths. It may also be difficult to specify the cause of death in cases where patients had other diseases coexisting with COVID-19, e.g., an advanced stage of cancer. Taking all this into account, it is very likely that the real number of deaths from COVID-19 is higher than the reported number of deaths, something that has been noticed in some countries, e.g., Italy^36,37^ and China^38^. It may well be that the numbers of both confirmed new cases and confirmed deaths are unreliable, yet no other data are available. Some models (e.g.^4^) are capable of estimating the true number of infections, but this involves making some additional assumptions and is based partly on the reporting of testing-dependent data. Additionally, the relationship between the true number of infections and the number of deaths has not been well studied to date and requires making several assumptions. Using the number of infections appears to be the easiest way of obtaining basic data on the COVID-19 infection dynamics in a given country, so long as one is aware that publicly shared data indicate the number of confirmed cases, not real infections, and takes this into account when interpreting the results.

In conclusion, the method based on fitting Johnson CDF curves to the cumulative number of confirmed cases is straightforward, well known and easy to use. It yields curves that are extremely well fitted to the data; thus, the basic parameters of COVID-19 infection dynamics obtained are easy to interpret and use in further statistical analyses by researchers from fields other than epidemiology, e.g., sociology, biology and ecology. While deepening our understanding of the COVID-19 pandemic, the Johnson CDF curve-fitting method may also be useful for making short-term predictions, although caution is advisable in such cases because the method is data dependent; if the data do not represent the true dimension of the epidemic, conclusions may be incomplete.

## Supplementary Information

Supplementary Information.

## Data Availability

The datasets generated during and/or analysed during the current study are available in the Dryad repository, https://doi.org/10.5061/dryad.f4qrfj6w9.
