# Supplementary material for: A simple method to describe the COVID-19 trajectory and dynamics in any country based on Johnson cumulative density function fitting
Source: Sci Rep. 2021 Sep 7;11:17744. doi: 10.1038/s41598-021-97285-5 (PMC8423734; doi:10.1038/s41598-021-97285-5)
Supplement: Supplementary file 1 — Supplementary Information. [file 41598_2021_97285_MOESM1_ESM.pdf]

Supplementary Materials for

A simple method to describe the COVID-19 trajectory and dynamics in any country based on Johnson cumulative density function fitting.

Adam M. Ćmiel*[1], Bogdan Ćmiel[2]

[1] Institute of Nature Conservation, Polish Academy of Sciences, al. A. Mickiewicza 33, 31-120 Kraków, Poland
[2] Faculty of Applied Mathematics, AGH University of Science and Technology, al. A. Mickiewicza 30, 30-059 Kraków, Poland

corresponding author: cmiel@iop.krakow.pl

Fitting Johnson cumulative density functions to cumulative epidemic waves

There is no strict definition of what is or is not an epidemic wave or phase. The intuitive definition of a pandemic wave traces the development of an epidemic over time and/or space. During an epidemic, the number of new cases of infection increases (often rapidly) to a peak and then falls (usually more gradually) until the epidemic wave is over. Each epidemic wave may be visualized by an epidemic curve (Fig. S1a). To visualize an epidemic curve, we put the number of cases on the vertical axis and the time unit on the horizontal axis. Another possible way of visualizing an epidemic wave is to place the cumulative number of cases on the vertical axis. In such cases, we obtain a cumulative epidemic curve (sigmoid shape instead of a "wave-like" shape; Fig. S1b). Nevertheless, the cumulative epidemic curve, even if it does not present the wave itself, describes the same epidemic wave or phase as the epidemic curve.

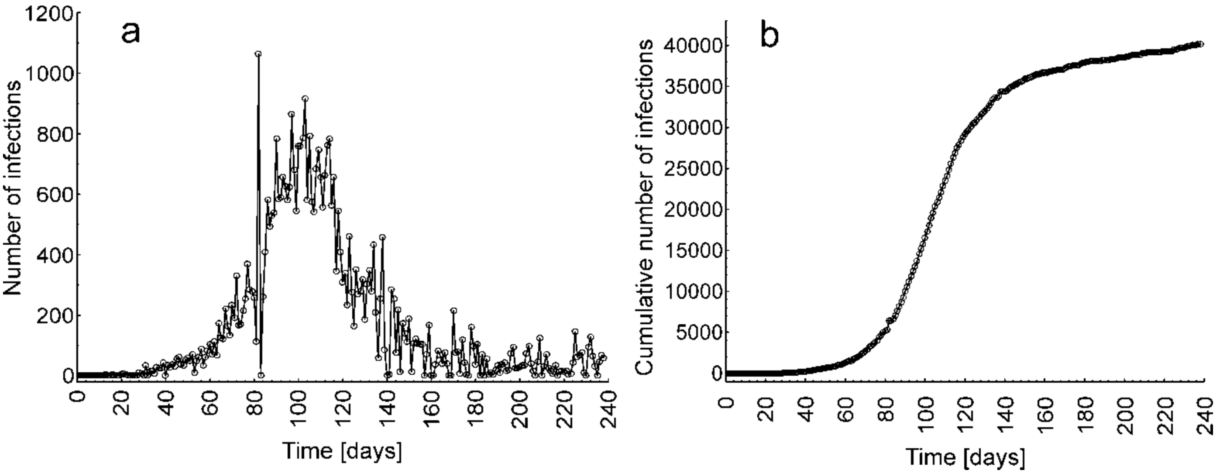

Figure S1. Examples of epidemic curves (**a**) and cumulative epidemic curves (**b**) describing the same infection wave in Afghanistan.

The method of fitting the Johnson cumulative density function to the epidemic wave is presented below.

Fitting the Johnson Cumulative Density Curve to one epidemic wave

Let us suppose that epidemic waves can be described by a five-parameter scaled Johnson unbounded CDF: scale parameter (*s*) and four moments – expected value (mean, *E*), standard deviation (σ), skewness (*S*) and kurtosis (*K*),

$$W(t)=s*F_{E,\sigma,S,K}(t) \qquad \text{(S1)},$$

where *t* is the time measured since the day of the beginning of the pandemic, and the function $F_{E,\sigma,S,K}$ is the Johnson unbounded (S_U) CDF with some parameters γ, δ, ξ, and λ assuming the mean, standard deviation, skewness and kurtosis to be equal to $E,\sigma,S,$ and *K*, respectively.

The method of fitting the Johnson CDF to the epidemic wave was presented on the data from Afghanistan. The fitting may be performed in 4 steps:

Step 1. Smoothing the raw data

It is advised to smooth the raw data before the curve fitting process (e.g., using a moving average), especially when one is trying to perform curve fitting when one full epidemic wave is not observed (ongoing wave of infections). Although smoothing did not affect the fitting process to the full epidemic wave, it may be useful when fitting it to the ongoing wave of infections because smoothing the data decreases the sensitivity of the numerical estimation method to changes in the starting point values (see sensitivity analysis), which, in this case, are more difficult to evaluate (see step 3). Moreover, smoothing makes the loss function more regular, so for the numerical algorithm, it is more difficult to confuse the local minimum of the loss function with the global minimum.

Step 2. Visualizing the epidemic wave using the cumulative epidemic curve

It is recommended that Johnson CDFs be fit to cumulative epidemic curves instead of fitting Johnson PDFs to the epidemic curves because the cumulative epidemic curve is a monotonic (nondecreasing) and smoother curve than the epidemic curve (Fig. S1a,b). In fact, the epidemic curve is a first derivative of the cumulative epidemic curve; thus, from estimation theory, it is already known that the estimation of derivatives is more difficult than the estimation of the curve itself. Moreover, considering the cumulative number of infections greatly reduces the random noise occurring in the daily number of infections (see Fig. S1).

Step 3. Finding starting points

The main advantage of using an alternative fitting method (using moments instead of the original shape coefficients) is that at least three starting points for coefficients *S*, *E*, and σ are intuitive and may be obtained visually from the cumulative epidemic curve (or at least, a range in which the values of the starting points are expected; Fig. S2).

When a full infection wave is observed, the value of the starting point for the scale parameter *s* may be evaluated as a value from the interval on the *y* axis, where the increase in the cumulative epidemic curve becomes low (in the case of Afghanistan, the *s* parameter was evaluated in approximately 40,000 infections; interval (S_L, S_U); Fig. S2). When only part of the infection wave is observed, the starting point may be evaluated as double the value of the cumulative epidemic curve in the moment of its fastest growth. However, this starting point may be very difficult to evaluate and highly underestimated when only the beginning of the infection wave is observed (also see Sensitivity analysis section which is presented later in this Supplementary Materials).

The value of the starting point for the *E* parameter may be evaluated visually from the cumulative epidemic curve, from the time interval (E_L, E_U), which contains the period of the fastest growth of the curve (Fig. S2) and which is usually indicated by the inflexion point. When the inflexion point is not visible, the starting point for the *E* parameter may be difficult to predict, and its value should be set to at least the maximum value on the time axis (e.g., if 80 days of the infection wave are observed and no inflexion point is visible, the value of *E* may be set to ≥80).

The value of the starting point for the σ parameter may also be evaluated visually from the cumulative epidemic curve as an (σ_L, σ_U) interval (Fig. S2). The lower bound value may be obtained as a time period between the moment of the beginning of the fast increase of the cumulative curve to the inflexion point, while the upper bound value may be obtained as a time period between the inflexion point and the moment where the cumulative curve starts to "flatten". When the inflexion point is not visible, the starting point of the σ parameter may be difficult to predict, and its value should be set at least as high as the time period from the beginning of the fast increase of the cumulative curve to the last observation.

Obtaining the starting point for skewness (*S*) and kurtosis (*K*) parameters is more difficult because it is not possible to evaluate their values visually. However, if $\sigma_L > \sigma_U$ is evaluated, it indicates that the starting point for the *S* parameter value is negative, while $\sigma_L = \sigma_U$ or $\sigma_L < \sigma_U$ indicates zero or positive values of the *S* parameter starting point, respectively. However, the sensitivity analysis of the algorithm to the changes in starting point values (see Sensitivity analysis) showed that when *s*, *E* and σ are properly selected, the estimation method is not sensitive to the changes in the values of the starting points *S* and *K*. Thus, in the presented examples, the starting points for the *S* and *K* parameters were set to *S*=1 and *K*=100.

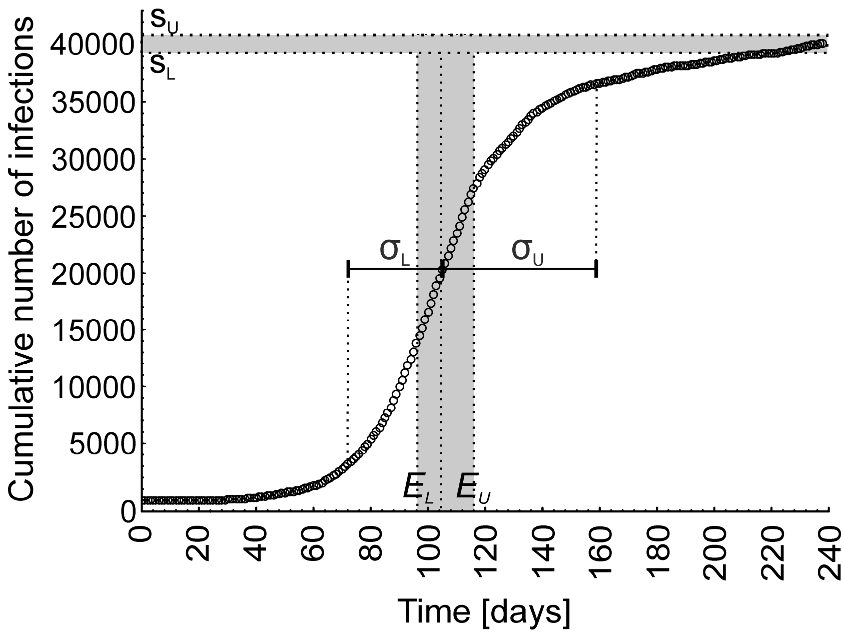

Figure S2. The general idea of the evaluation of the intervals where the values of starting points for parameters *s*, *E* and σ are expected is to visually analyse the cumulative epidemic curve.

In conclusion, by visually analysing the cumulative epidemic curve from Afghanistan, starting point values may be evaluated at $s \in (39000, 41000)$, $E \in (96, 116)$, and $\sigma \in (30, 60)$.

Step 4. Fitting using alternative sets of starting points

After visually selecting an initial set of starting points, it is recommended to perform fitting using other sets of starting points from the evaluated intervals. The results of 15 Johnson CDFs fitting to the cumulative epidemic curve from Afghanistan are presented in Table S1.

Table S1. Example of 10 Johnson curve fittings to the infection wave reported in Afghanistan using different sets of starting points.

Selected starting points					Estimated curve parameters					R^2
s	*E*	σ	*S*	*K*	*s*	*E*	σ	*S*	*K*	
39000	90	40	1	100	40005.49	111.081	41.5161	3.53541	51.3501	0.99980
39000	105	40	1	100	40005.49	111.081	41.5161	3.53541	51.3501	0.99980
39000	111	41	3.5	51	40005.49	111.081	41.5161	3.53541	51.3501	0.99980
39000	120	40	1	100	40005.49	111.081	41.5161	3.53541	51.3501	0.99980
39000	140	40	1	100	40005.49	132.712	51.3125	3.12841	21.3694	0.9456
39000	100	60	1	100	40005.49	111.081	41.5161	3.53541	51.3501	0.99980
39000	120	80	1	100	40005.49	111.081	41.5161	3.53541	51.35	0.99980
39000	110	50	5	100	40005.49	111.081	41.5162	3.53541	51.3503	0.99980
39000	120	10	3	20	40005.49	111.081	41.5161	3.53541	51.3501	0.99980
39000	100	50	0	0	38309.19	104.56	24.3863	0	0	0.9980
41000	105	40	1	100	40007.6	111.098	41.5949	3.57839	52.7383	0.99978
41000	111	41	3.5	51	40007.6	111.098	41.5949	3.57839	52.7383	0.99978
41000	120	40	1	100	40007.6	111.098	41.5949	3.57839	52.7383	0.99978
40000	111	45	1	60	40007.6	111.098	41.5949	3.57839	52.7383	0.99978
40000	111	40	1	100	40007.6	111.098	41.5949	3.57839	52.7383	0.99978

The best fitted curve should be used as a final estimation result. In the case of Afghanistan, the best fitted curve (R^2=0.9998) parameters are s=40005.49, E=111.081, σ=41.5161, S=3.53541 and K=51.3501. This curve can then be used to calculate the basic parameters describing the dynamics of the infection wave: the day the infection wave started ($Q_{2.5\%}$), the day the infection wave ended ($Q_{97.5\%}$), the day that half of the total percentage of infections during a given wave were reached ($Q_{50\%}$), the day when the infection wave peaked (M), the duration of the wave (T), the duration of the wave increase (t_i), the duration of the wave decrease (t_d), and wave asymmetry (A; see eqs. 2-11 in the main text).

Adding more Johnson curves to fit more epidemic waves

Let us suppose that n epidemic waves (W) occurred in a given country and that each epidemic wave can be described by a five-parameter scaled Johnson unbounded CDF (see eq. S1). Thus, the trajectory of the epidemic $E(t)$ can be described by

$$E(t)=W_{1,n}(t)+ W_{2,n}(t)+\ldots+ W_{n,n}(t) \qquad \text{(S2)}$$

Below, fitting multiple Johnson CDFs to fit the trajectory of the epidemic was presented for examples from Australia and Poland.

Example 1. Fitting Johnsons CDFs to the data from Australia

After smoothing the data, the epidemic trajectory in Australia was visualized as a cumulative epidemic curve. By visually analysing the cumulative epidemic wave, one can clearly distinguish two epidemic waves, which suggests that the epidemic trajectory in Australia can be described by two Johnson CDFs (Fig. S3a):

$$E(t)=W_{1,2}(t)+W_{2,2}(t) \qquad \text{(S3)}$$

The parameters for each curve were obtained as described above (steps 3-4). Only the results for the best fitted curves are presented in this example. Moreover, for the sake of exemplification, the results of fitting only one Johnson CDF and three Jonson CDFs to the cumulative epidemic curve from Australia were also presented (Table S2, Fig. S3b).

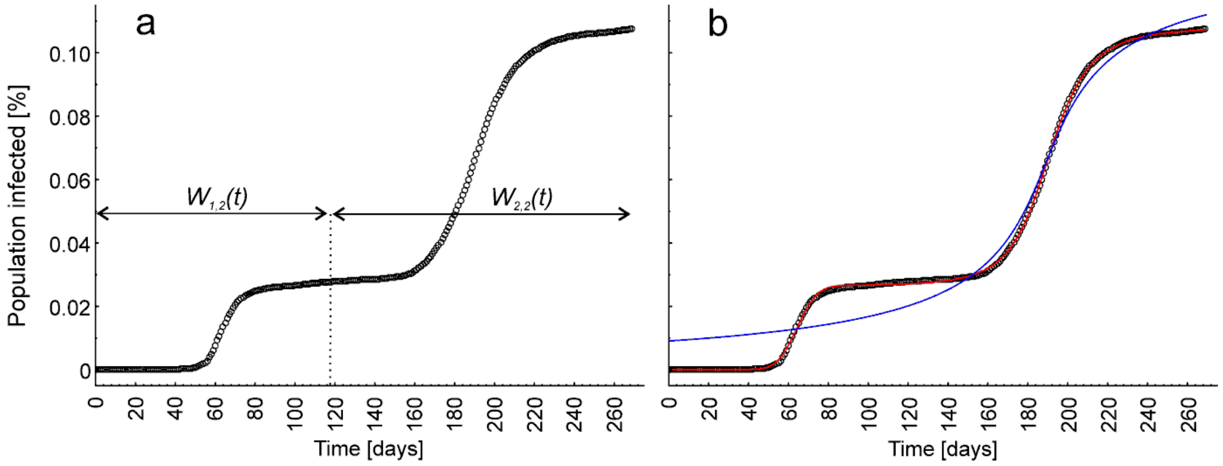

Figure S3. (**a**) The cumulative epidemic curve from Australia with easily distinguishable epidemic waves and (**b**) one fitted Johnson CDF (blue line) and two Johnson CDFs (red line) describing the epidemic trajectory in Australia.

Table S2. The results of fitting one, two and three Johnson CDFs to the cumulative epidemic curve from Australia.

Number of Johnson CDFs fitted	Fitted curve	R^2
1	$E(t)=0.130002*F_{188.372,1173.77,0.00208045,6,49738e+007}(t)$	0.96813
2	$E(t)=0.027695*F_{67.0193,15.1412,4.91014,91.1291}(t)+$ $+0.0797208*F_{191.801,22.0842,0.650094,5.48317}(t)$	0.99996
3	$E(t)=0.02692*F_{66.2612,12.5528,3.40836,39.7692}(t)+$ $+0.01277*F_{240.259, 80.4446,0.331707, 0.0043}(t)+$ $+0.0724*F_{190.511,17.6172,0.191,1.13908}(t)$	0.99996

The results showed that the cumulative epidemic curve from Australia was best described by fitting two Johnson CDFs (Table S2; Fig. S3b). One Johnson CDF was far more poorly fitted than the two Johnson CDFs (Table S2, Fig. S3b), whereas fitting three Johnson CDFs did not improve the fit; thus, adding an additional five parameters is pointless.

Example 2. Fitting Johnsons CDFs to the data from Poland

After smoothing the data, the epidemic trajectory in Poland was visualized as a cumulative epidemic curve. However, unlike in Australia, it is not easy to visually obtain the number of epidemic waves (Fig. S4a). The cumulative epidemic curve in Poland may either be described by one epidemic wave

$$E(t)=W_{1,1}(t) \qquad \text{(S4)},$$

two epidemic waves

$$E(t)=W_{1,1}(t)+W_{2,2}(t) \qquad \text{(S5)},$$

or three epidemic waves

$$E(t)=W_{1,3}(t)+W_{2,3}(t) +W_{3,3}(t) \qquad \text{(S6)}.$$

The parameters for each curve were obtained as described above (steps 3-4). Only the results for the best fitted curves are presented in this example. Moreover, for the sake of example, the results of fitting four Johnson CDFs to the cumulative epidemic curve from Poland were also presented (Table S3, Fig. S4b).

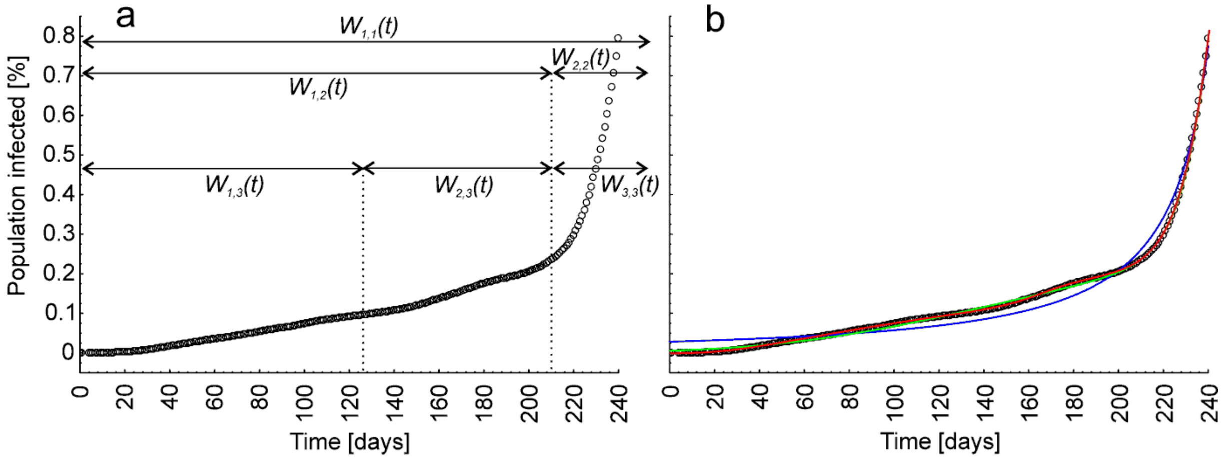

Figure S4. (**a**) The cumulative epidemic curve from Poland, which may be described by one, two or three epidemic waves, and (**b**) one fitted Johnson cumulative density function (blue line), two Johnson cumulative density functions (green line) and three Johnson cumulative density functions (red line) describing the epidemic trajectory in Poland.

Table S3. The results of fitting one, two, three and four Johnson cumulative density functions to the cumulative epidemic curve from Poland.

Number of Johnson CDFs fitted	Fitted curve	R^2
1	$E(t)= 2.47592*F_{247.841,155.074,0.00298371,322200}(t)$	0.96676
2	$E(t)=0.223036*F_{137.599,72.0234,0.00115829,0.00168398}(t)+$<br>$+2.71657*F_{259.966,32.7988,2.75232,25.4339}(t)$	0.99789
3	$E(t)=0.143335*F_{112.426,65.9492,0.904095,0.514262}(t)+$<br>$+0.0658981*F_{166.643,25.4159,0.00640294,13.5111}(t)+$<br>$+2.39085*F_{250.757,18.1073,0.00110578,1.72038}(t)$	0.99981
4	$E(t)= 0.0766458*F_{115.953,41.487,1.9145,7.16402}(t)+$<br>$+0.0713785*F_{165.56,16.8285,0.00341615, 0.00525927}(t)+$<br>$+2.35334*F_{250.217,18.5738, 0.00903355, 2.37484}(t)+$<br>$+0.0414949*F_{44.7297, 15.6349, 0.00632781, 0.000867302}(t)$	0.99982

The results showed that the cumulative epidemic curve from Poland was best described by fitting three Johnson CDFs (Table S3; Fig. S4b). Fitting one and two CDFs resulted in lower R^2 values compared to the R^2 of three Johnson CDFs, whereas adding the fourth Johnson CDF improved the fit only by 0.00001, suggesting that it is not worth adding an additional five parameters to the estimate (Table S3).

Sensitivity analysis

Sensitivity analysis was performed to check 1) the sensitivity of the numerical algorithm to data perturbation, 2) the sensitivity of the algorithm to changes in selected starting point values, 3) the sensitivity of the fitted curve to the change in the parameter value and 4) the influence of smoothing the raw data on the sensitivity of the algorithm to changes in selected starting point values.

Sensitivity of the best fitted Johnson curve to data perturbations and errors

To check the sensitivity of the best fitted Johnson CDF to perturbations on data, we have to decide what type of data perturbation should be considered. Since the mechanism of generating the real cumulative epidemic curve is unknown, and in fact may also be very complex, a problem of fitting a scaled Johnson CDF to discretized and censored data generated from a fixed Johnson distribution was considered. This is performed to check the sensitivity of the numerical algorithm to data perturbation depending on the censoring time point and the sample size. In this case, it is possible to obtain a 95% confidence interval for estimated curve parameters and a 95% confidence area for the curve using parametric bootstrap methods.

The observations were generated from the fixed Johnson cumulative distribution on the time axis using the E=111.081, σ=41.5161, S=3.53541and K=51.35 parameter values for three sample sizes (total number of infections; s_1=20,000; s_2=100,000; ands_3=500,000). Next, the number of observations on consecutive days was counted and presented as a cumulative curve of observations (cumulative epidemic curve). For each sample generated in this way, the expected value of the number of observations (infections) lies on our fixed Johnson curve, but for each sample, those numbers are slightly different (perturbed). For each generated bootstrap sample, a new Johnson curve was fitted:

$$W_k(t)=s_k*F_{111,41,3.5,51}(t),\ k=1,2,3 \qquad \text{(S7)},$$

using only the data up to a certain day. The most interesting time points of censoring are:

- [0,72], before the fastest growth of the cumulative curve (when the inflexion point of the cumulative epidemic curve is not visible)
- [0, 96], just before, but close to the fastest growth of the cumulative curve (very close to the inflexion point of the cumulative epidemic curve)
- [0, 120], after the fastest growth of the cumulative curve (when the inflexion point of the cumulative epidemic curve is visible)

Basic statistics and 95% confidence intervals for parameters of fitted Johnson CDFs are presented in Table S4, and the 95% confidence area for the curve depending on the sample size and censoring point is presented in Fig. S5.

Table S4. Basic statistics for estimated parameters of Johnson CDFs depending on sample size and censoring point, showing the sensitivity of the algorithm on data perturbations.

Sample size	Censoring time point (Observed days)	Parameter	Mean	SD	2.5% Quantile	97.5% Quantile	Median	"True" (fixed) value
20 000	72	*s*	37881.19	25071.22	4003.23	87496.8	39091.6	20000
		E	119.4992	19.20693	77.4819	173.232	116.394	111
		σ	57.95834	51.12783	18.2832	189.418	34.30355	41
		S	4.523104	4.302203	0.189479	14.7557	2.85422	3.5
		K	202.6737	331.5898	11.569	1172.38	47.16355	51
	96	*s*	23314.69	10559.55	13331.5	50881.1	19126.8	20000
		E	121.3266	31.96079	95.0675	201.996	108.1995	111
		σ	63.56083	57.09327	24.2592	205.351	39.0404	41
		S	3.787686	3.422118	0.01	10.4233	2.92576	3.5
		K	108.5343	143.0198	10.9363	447.387	34.879	51
	120	*s*	20186.68	839.1431	18576.3	21564.7	20155.9	20000
		E	111.4958	3.522126	104.619	117.818	111.188	111
		σ	43.40251	6.766972	30.7343	59.0294	42.18555	41
		S	3.749606	1.086217	1.47228	6.47476	3.64146	3.5
		K	61.52343	31.73622	15.5444	171.337	52.465	51
100 000	72	*s*	135414.5	74460.42	46316.7	342713	116609.5	100000
		E	112.2878	8.177635	96.8123	129.483	113.682	111
		σ	42.8125	11.9638	24.3561	62.5007	38.358	41
		S	3.638708	1.082213	1.49648	5.35217	3.187395	3.5
		K	60.93808	26.46275	24.0741	125.754	56.68605	51
	96	*s*	104653.7	20769.21	77648	153699	103038.5	100000
		E	112.7787	10.67289	99.9011	142.146	111.4815	111
		σ	45.03618	15.15036	29.4428	93.5803	41.10035	41
		S	3.474125	1.595832	1.48679	7.11799	3.60621	3.5
		K	57.96746	36.18965	20.3824	178.907	53.34795	51
	120	*s*	99619.43	1731.655	95441.4	102761	99402.8	100000
		E	110.2936	1.434343	107.052	113.146	110.058	111
		σ	40.96818	2.640203	35.116	46.6521	40.2402	41
		S	3.428888	0.430988	2.47405	4.31246	3.33904	3.5
		K	49.47903	10.19388	31.0023	72.3694	48.0193	51
500 000	72	*s*	569561.8	261285.3	257366	1238820	472917.5	500000
		E	113.4321	9.326847	98.1453	128.951	116.235	111
		σ	46.24778	9.933583	29.4899	59.1436	51.9388	41
		S	3.813146	1.073631	1.49465	4.94355	4.6098	3.5
		K	60.87341	20.50203	23.429	92.6758	69.62885	51
	96	*s*	495591.2	52066.23	413173	592510	482470.5	500000
		E	110.1882	5.428912	101.357	120.08	108.855	111
		σ	41.27961	7.375042	30.0752	54.3512	39.4843	41
		S	3.356778	0.930904	1.56296	4.86677	3.21771	3.5
		K	50.55106	18.27404	24.0876	87.1048	46.27765	51
	120	*s*	499632.8	3509.042	493599	506644	499467	500000
		E	110.5131	0.590449	109.498	111.645	110.483	111
		σ	41.39574	1.150893	39.2865	43.407	41.2768	41
		S	3.506785	0.193949	3.1271	3.83708	3.49625	3.5
		K	50.75436	4.64953	41.6607	58.8116	50.7473	51

The results showed that the fitted Johnson CDF was very sensitive to data perturbation and errors, but only when it was fitted using just 30% of the available data (72 days of the ongoing infection wave, far before the cumulative epidemic curve inflexion point). When 40% of the available data (96 days of the ongoing infection wave, just before the cumulative epidemic curve inflexion point) was used to fit the Johnson CDF, its sensitivity to data perturbation greatly decreased, while it was hardly sensitive to data perturbation when 50% of the data (120 days of the ongoing wave, after the inflexion point of the cumulative epidemic curve) was used in the estimation (Table S4; Fig. S5)

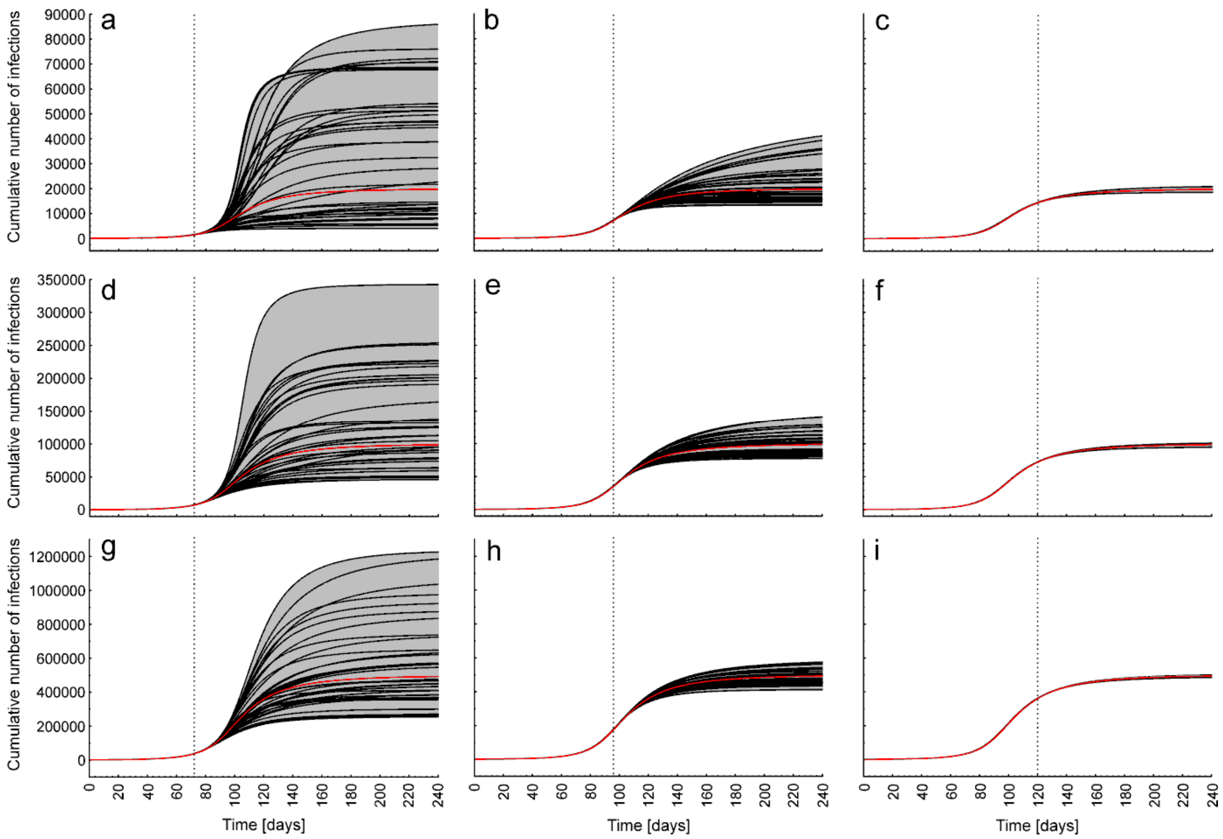

Figure S5. Confidence areas (95%; grey) for Johnson CDF (red line) showing the sensitivity of the numerical algorithm to data perturbation, depending on the sample size and censoring point: (**a**) sample size = 20,000, censoring point at 72nd day; (**b**) sample size = 20,000, censoring point at 96th day; (**c**)sample size = 20,000, censoring point at 120th day; (**d**) sample size = 100,000, censoring point at 72nd day; (**e**) sample size = 100,000, censoring point at 96th day; (**f**)sample size = 100,000, censoring point at 120th day; (**g**) sample size = 500,000, censoring point at 72nd day; (**h**) sample size = 500,000, censoring point at 96th day; and (**i**) sample size = 500,000, censoring point at 120th day.

Sensitivity analysis of the algorithm used to changes in selected starting point values

The sensitivity of the algorithm to the changes in the values of the selected starting points was determined on the data from Afghanistan using the parameters of the best fitted Johnson CDF. Default values for the starting points were set to s=0.103, E=111, σ=41, S=3.5, and K=51. Then, the sensitivity of the algorithm to the changes in the value of a given starting point was tested by performing multiple fitting, each time changing the value of a given starting point, while the values of the other starting points were set at their default values, to check if the final value of the estimated parameters would change, depending on the value of the starting point. The results of the sensitivity analysis of the algorithm used to the changes in values of selected starting points are presented in Table S5.

The results showed that the algorithm used is hardly sensitive to the selection of the starting point values. The only cases when the estimated curve was not correctly fitted to the data, which is visible by much lower R^2 values, were when the starting point for the E parameter was set at 250 and 300, which is over 2 times higher than its optimal value, and when the S parameter starting value was set to 0 (Table S5).

Table S5. The results of the sensitivity analysis of the algorithm used to the changes in values of selected starting points.

Parameter tested	Starting point value	R^2	Estimated parameters				
			s	*E*	*σ*	*S*	*K*
s	0.01	0.99979638	0.102767	111.081	41.5161	3.53541	51.35
	0.02	0.99979638	0.102767	111.081	41.5161	3.53541	51.35
	0.033	0.99979638	0.102767	111.081	41.5161	3.53541	51.35
	0.05	0.99979638	0.102767	111.081	41.5161	3.53541	51.35
	0.103	0.99979638	0.102767	111.081	41.5161	3.53541	51.35
	0.2	0.99979638	0.102767	111.081	41.5161	3.53541	51.35
	0.5	0.99979638	0.102767	111.081	41.5161	3.53541	51.35
	1.0	0.99979638	0.102767	111.081	41.5161	3.53541	51.35
E	11	0.99979638	0.102767	111.081	41.5161	3.5354	51.35
	22	0.99979638	0.102767	111.081	41.5161	3.5354	51.35
	37	0.99979638	0.102767	111.081	41.5161	3.5354	51.35
	56	0.99979638	0.102767	111.081	41.5161	3.5354	51.35
	60	0.99979638	0.102767	111.081	41.5161	3.5354	51.35
	70	0.99979638	0.102767	111.081	41.5161	3.53541	51.35
	80	0.99979638	0.102767	111.081	41.5161	3.5354	51.35
	90	0.99979638	0.102767	111.081	41.5161	3.53541	51.35
	100	0.99979638	0.102767	111.081	41.5161	3.5354	51.35
	111	0.99979638	0.102767	111.081	41.5161	3.53541	51.35
	120	0.99979638	0.102767	108.619	41.5161	3.53541	51.3501
	130	0.99979638	0.102767	108.619	41.5161	3.53541	51.35
	140	0.99979638	0.102767	108.619	41.5161	3.53541	51.35
	150	0.99979638	0.102767	108.619	41.5161	3.5354	51.35
	200	0.99979638	0.102767	111.081	41.5161	3.53541	51.35
	250	0.91969016	0.192534	222.367	178.257	2.54486	13.3597
	300	0.89827058	0.249301	268.221	177.402	1.18206	1.39523
σ	10	0.99979638	0.102767	111.081	41.5161	3.5354	51.35
	20	0.99979638	0.102767	111.081	41.5161	3.53541	51.35
	30	0.99979638	0.102767	111.081	41.5161	3.53541	51.35
	41	0.99979638	0.102767	111.081	41.5161	3.53541	51.35
	50	0.99979638	0.102767	111.081	41.5161	3.53541	51.35
	60	0.99979638	0.102767	111.081	41.5161	3.53541	51.35
	70	0.99979638	0.102767	111.081	41.5161	3.5354	51.35
	80	0.99979638	0.102767	111.081	41.5161	3.53541	51.35
	90	0.99979638	0.102767	111.081	41.5161	3.53541	51.35
	100	0.99979638	0.102767	111.081	41.5161	3.53541	51.3501
	300	0.99979638	0.102767	111.081	41.5161	3.53541	51.3501
S	-4	0.99979638	0.102767	111.081	41.5161	3.53541	51.35
	-3	0.99979638	0.102767	111.081	41.5161	3.53541	51.35
	-2	0.99979638	0.102767	111.081	41.5161	3.5354	51.35
	-1	0.99979638	0.102767	111.081	41.5161	3.53541	51.3501
	0	0.99857501	0.0990974	104.753	28.7334	0	4.68877
	1	0.99979638	0.102767	111.081	41.5161	3.53541	51.35
	2	0.99979638	0.102767	111.081	41.5161	3.53541	51.35
	3	0.99979638	0.102767	111.081	41.5161	3.53541	51.35
	3.5	0.99979638	0.102767	111.081	41.5161	3.53541	51.35
	4	0.99979638	0.102767	111.081	41.5161	3.53541	51.3501
K	15	0.99979638	0.102767	111.081	41.5161	3.53541	51.3501
	30	0.99979638	0.102767	111.081	41.5161	3.53541	51.35
	40	0.99979638	0.102767	111.081	41.5161	3.53541	51.3501
	51	0.99979638	0.102767	111.081	41.5161	3.53541	51.35
	60	0.99979638	0.102767	111.081	41.5161	3.53541	51.35
	70	0.99979638	0.102767	111.081	41.5161	3.53541	51.35
	80	0.99979638	0.102767	111.081	41.5161	3.53541	51.35
	100	0.99979638	0.102767	111.081	41.5161	3.53541	51.3501

Sensitivity of the fitted curve to the change in the parameter value

The sensitivity of the fitted curve to the changes in the value of parameters was determined on the data from Afghanistan. The default values for the parameters were set to *s*=0.102767, *E*=111.081, *σ*=41.5161, *S*=3.53541, and *K*=51.3501. Then, the sensitivity of the fitted curve to the changes in the value of a given parameter was tested by calculating the coefficients of determination (R^2), each time changing the value of a given parameter, while the values of the other parameters were set at their default values to determine how the R^2 will change, depending on the value of the given parameter. The results of the sensitivity analysis of the algorithm used to the changes in values of selected starting points are presented in Table S6.

The Johnson CDF curve fitted to the cumulative epidemic wave from Afghanistan was the most sensitive to the changes in the values of the *s* and *E* parameters. Changing the value of these parameters by more than ±5% resulted in a relatively high decrease in the R^2 value, whereas for other parameters, the R^2 value was still higher than 0.99, even after changing the value of the parameter by ±25% (Table S6).

Table S6. The results of the sensitivity analysis of the Johnson CDF (presented as R^2 values) depending on the percentage change in the value of a given parameter.

	Percentage change in the parameter value										
	-25%	-20%	-15%	-10%	-5%	0%	+5%	+10%	+15%	+20%	+25%
s	0.826486	0.887229	0.934915	0.969544	0.991115	0.9998	0.995086	0.977486	0.946829	0.903114	0.846342
E	0.812395	0.876832	0.929151	0.967984	0.991716	0.9998	0.991697	0.967713	0.928671	0.875900	0.811015
σ	0.993674	0.995989	0.997715	0.998895	0.999572	0.9998	0.999556	0.998931	0.997932	0.996587	0.994920
S	0.998641	0.998989	0.999292	0.999542	0.999713	0.9998	0.999699	0.999396	0.998744	0.997497	0.995096
K	0.999006	0.999353	0.999569	0.999696	0.999761	0.9998	0.999767	0.999728	0.999671	0.999600	0.999517

The influence of smoothing the raw data on the sensitivity of the algorithm to changes in selected starting point values

The sensitivity of the algorithm to the changes in the values of the selected starting points was determined on the raw and smoothed (5-day moving average) data from Switzerland using the parameters of the best fitted Johnson CDF. Default values for the starting points were set to s_1=0.103, E_1=111, $σ_1$=41, S_1=3.5, and K_1=51 for the first wave of infections, to s_2=0.103, E_2=111, $σ_2$=41, S_2=3.5, and K_2=51 for the second wave of infections, and to s_3=0.103, E_3=111, $σ_3$=41, S_3=3.5, and K_3=51 for the third wave of infections. Then, the sensitivity of the algorithm to the changes in the value of an E_3 parameter was tested by performing multiple fitting, each time changing the value of an E_3 parameter starting point, while the values of the other starting points were set at their default values to check if the final value of the estimated parameters would change, depending on the value of the starting point and smoothing the data.

The results showed that when fitting Johnson CDF to the ongoing wave, smoothing the raw data makes the numerical algorithm less sensitive to the changes in the values of the selected starting points (Table S7).

Table S7. The results of the sensitivity analysis of the used algorithm to the changes in values of the selected starting point (E_3), performed on nonsmoothed and smoothed (5-days moving average) data from Switzerland.

Data used	Wave number	Starting point value for *E*	Estimated parameters					R^2
			s	*E*	*σ*	*S*	*K*	
Nonsmoothed	1	35	0.346415	36.8513	12.9059	1.09336	2.52266	0.9997
	2	209	0.443446	209.623	54.0862	0.257488	5.1684	
	3	232	0.220582	232.239	7.01495	0.00727957	108.8	
	1	35	0.345336	36.8149	12.7772	1.06679	2.34056	0.9997
	2	209	0.444303	209.523	56.1002	0.315109	6.87226	
	3	235	0.220408	232.269	6.14191	0.0966419	44.342	
	1	35	0.349781	31.417	12.0022	0.347605	51.7844	0.9794
	2	209	0.451029	208.885	51.5855	0.952251	77.5894	
	3	240	0.272046	239.068	5.64267	0.750697	1.02849	
Smoothed (5-days moving average)	1	35	0.346704	36.8503	13.0004	1.07072	2.45471	0.9997
	2	209	0.450417	209.331	52.0351	0.07506	4.15864	
	3	232	0.207681	232.148	4.75103	0.00360	4.04213	
	1	35	0.346591	36.8564	12.9996	1.07328	2.46237	0.9997
	2	209	0.442682	208.922	52.9049	0.161177	4.76164	
	3	235	0.21492	232.182	5.53929	0.0414654	11.0521	
	1	35	0.347763	36.8964	13.14	1.0947	2.64168	0.9997
	2	209	0.454353	209.671	50.1219	0.000005	3.03358	
	3	240	0.204025	232.143	4.56713	0.00146299	3.19946	

Fitted Johnson cumulative density functions

Johnson cumulative density functions fitted to the data obtained for 80 countries on six continents are shown in Figs. S6-S11 (Africa, Fig. S6; Asia, Fig. S7; Europe, Fig. S8; North America, Fig. S9; Oceania, Fig. S10; and South America, Fig. S11). The formulas and R^2 values of each fitted curve are listed in Table S8. The basic parameters of the first infection wave dynamics (S, P_{inf}, $Q_{2.5\%}$, $Q_{50\%}$, $Q_{97.5\%}$; M, t_i, t_d, T, and A) calculated using Johnson CDFs fitted to the data obtained for 80 countries on six continents are listed in Table S9.

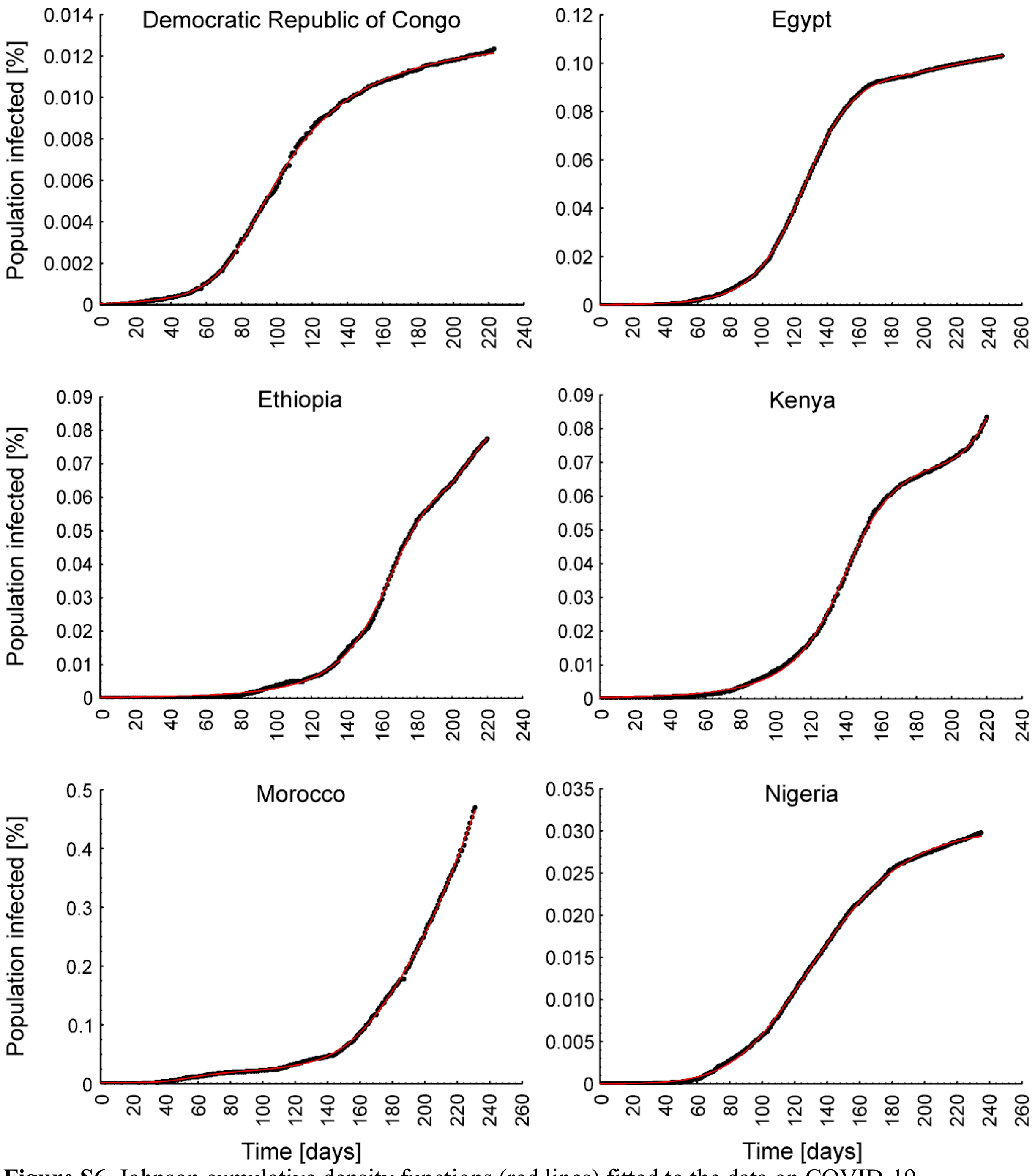

Figure S6. Johnson cumulative density functions (red lines) fitted to the data on COVID-19 trajectories (cumulative epidemic curves) in African countries.

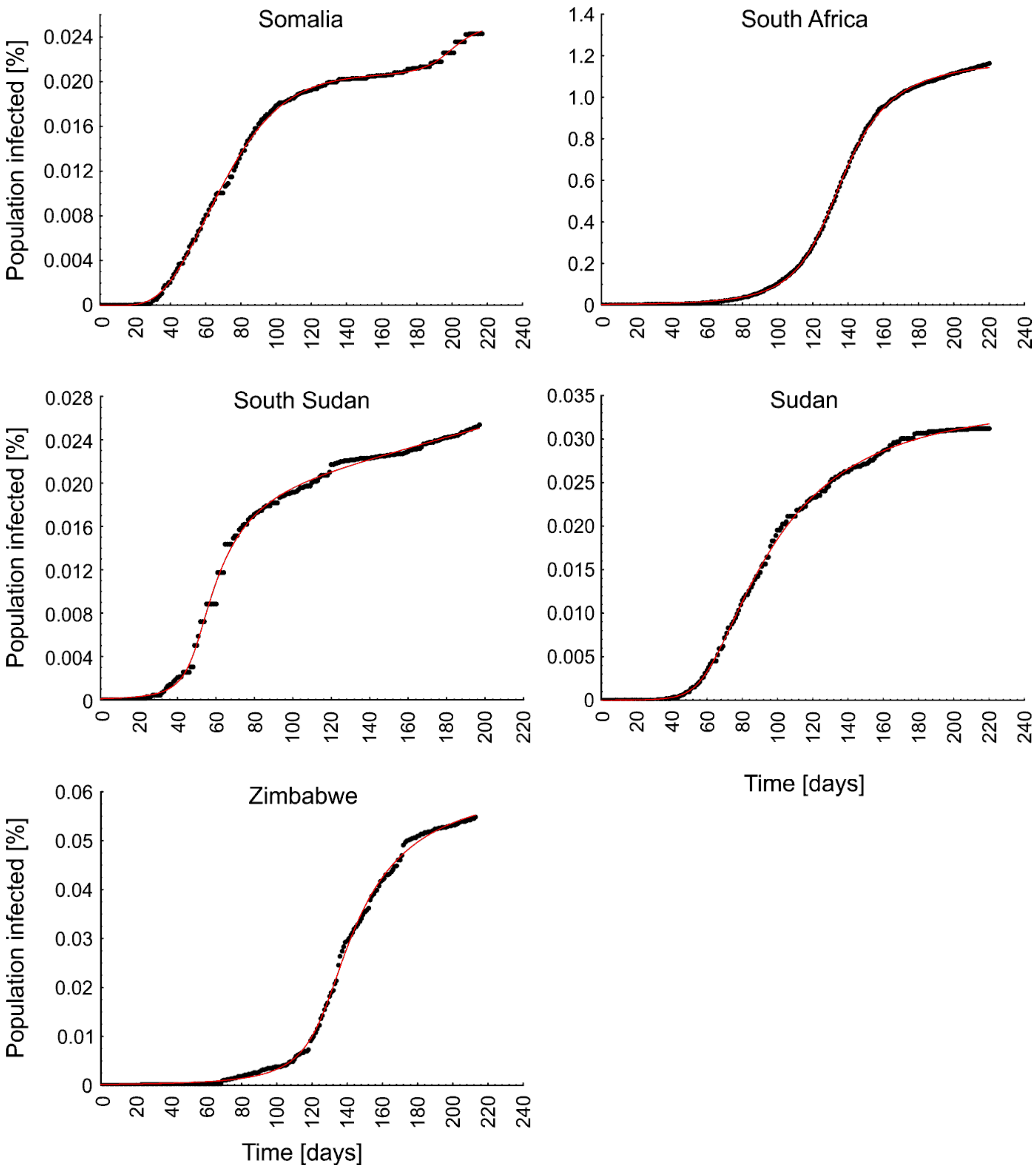

Figure S6 continued. Johnson cumulative density functions (red lines) fitted to the data on COVID-19 trajectories (cumulative epidemic curves) in African countries.

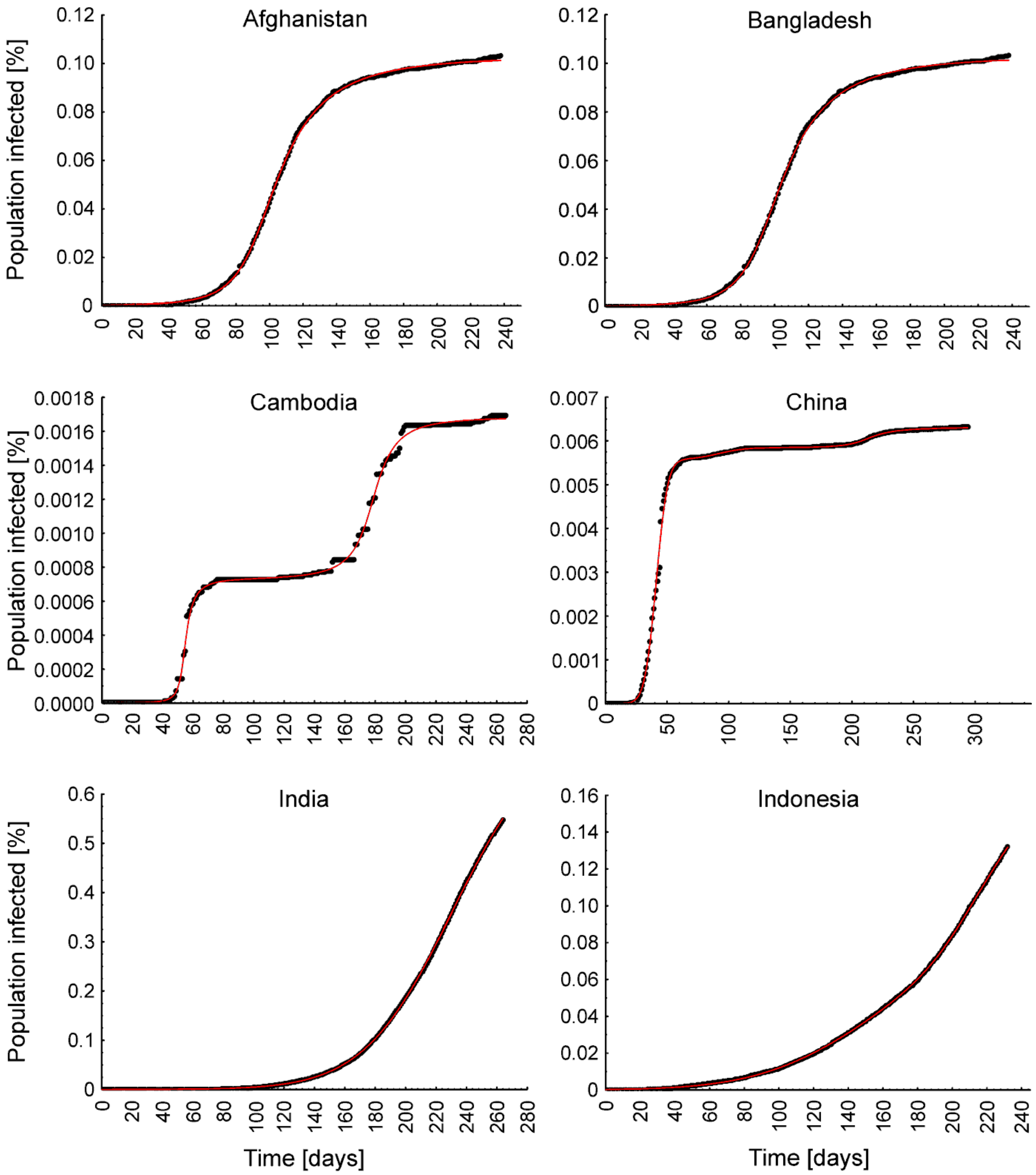

Figure S7. Johnson cumulative density functions (red lines) fitted to the data on COVID-19 trajectories (cumulative epidemic curves) in Asian countries.

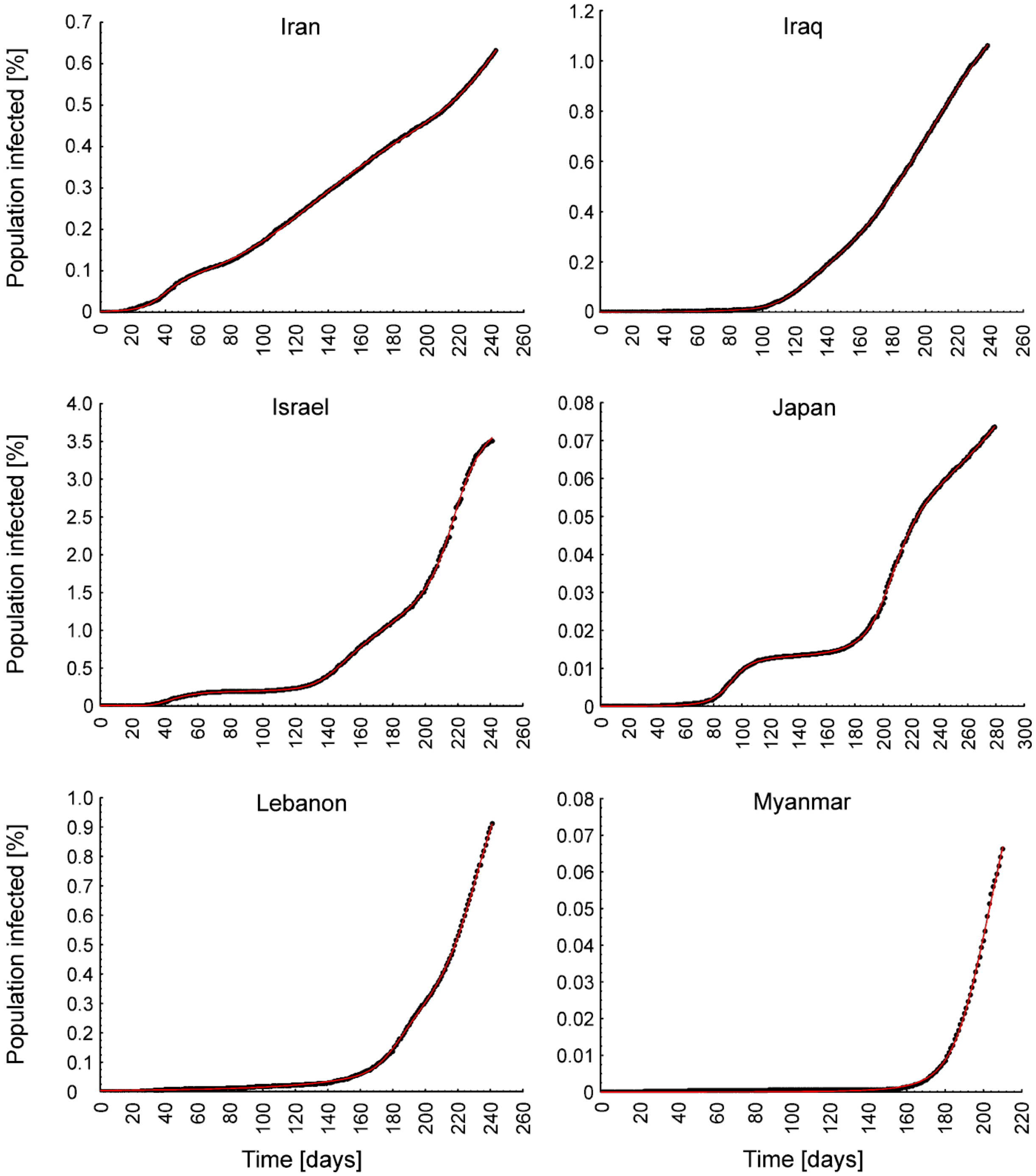

Figure S7 continued. Johnson cumulative density functions (red lines) fitted to the data on COVID-19 trajectories (cumulative epidemic curves) in Asian countries.

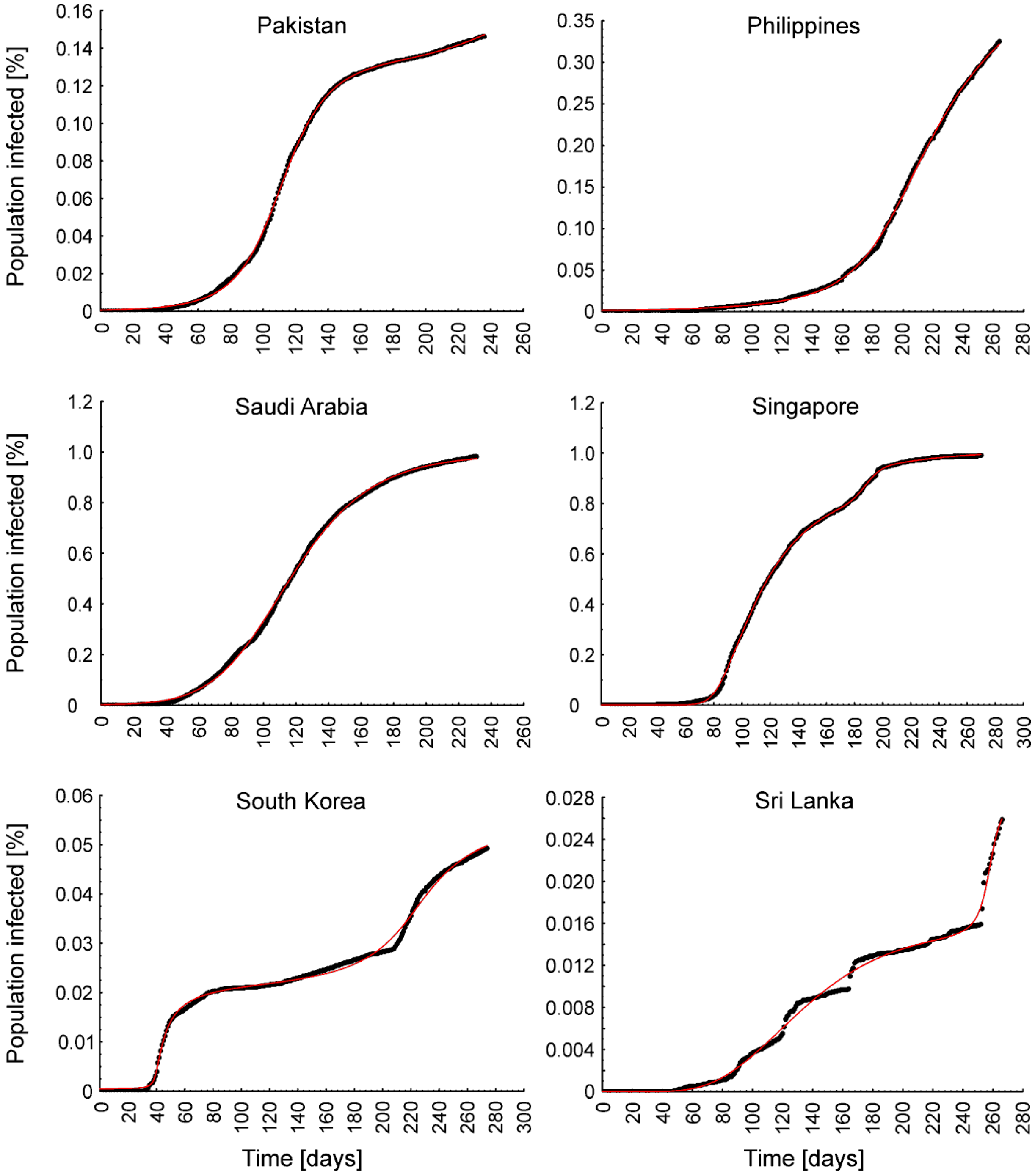

Figure S7 continued. Johnson cumulative density functions (red lines) fitted to the data on COVID-19 trajectories (cumulative epidemic curves) in Asian countries.

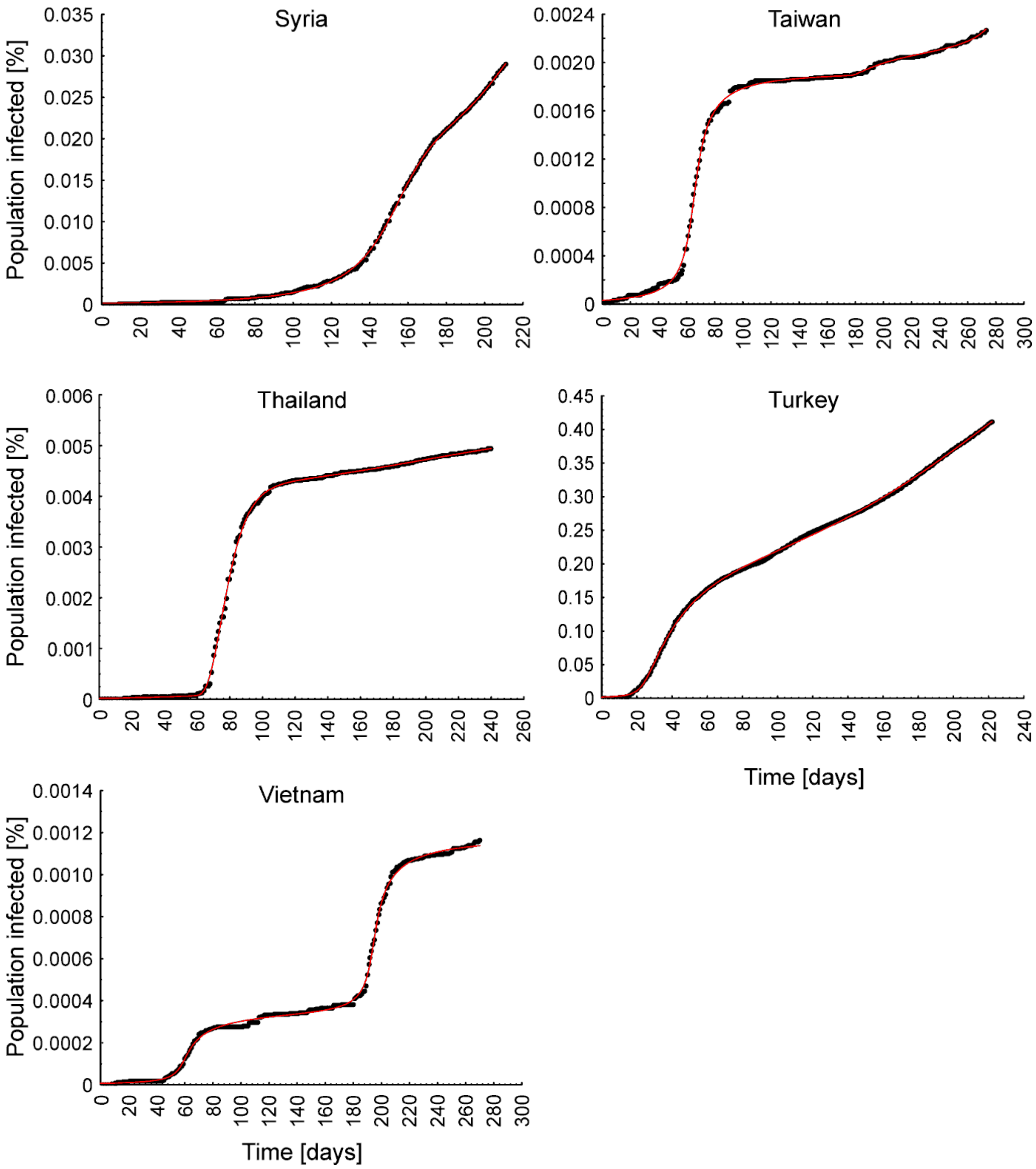

Figure S7 continued. Johnson cumulative density functions (red lines) fitted to the data on COVID-19 trajectories (cumulative epidemic curves) in Asian countries.

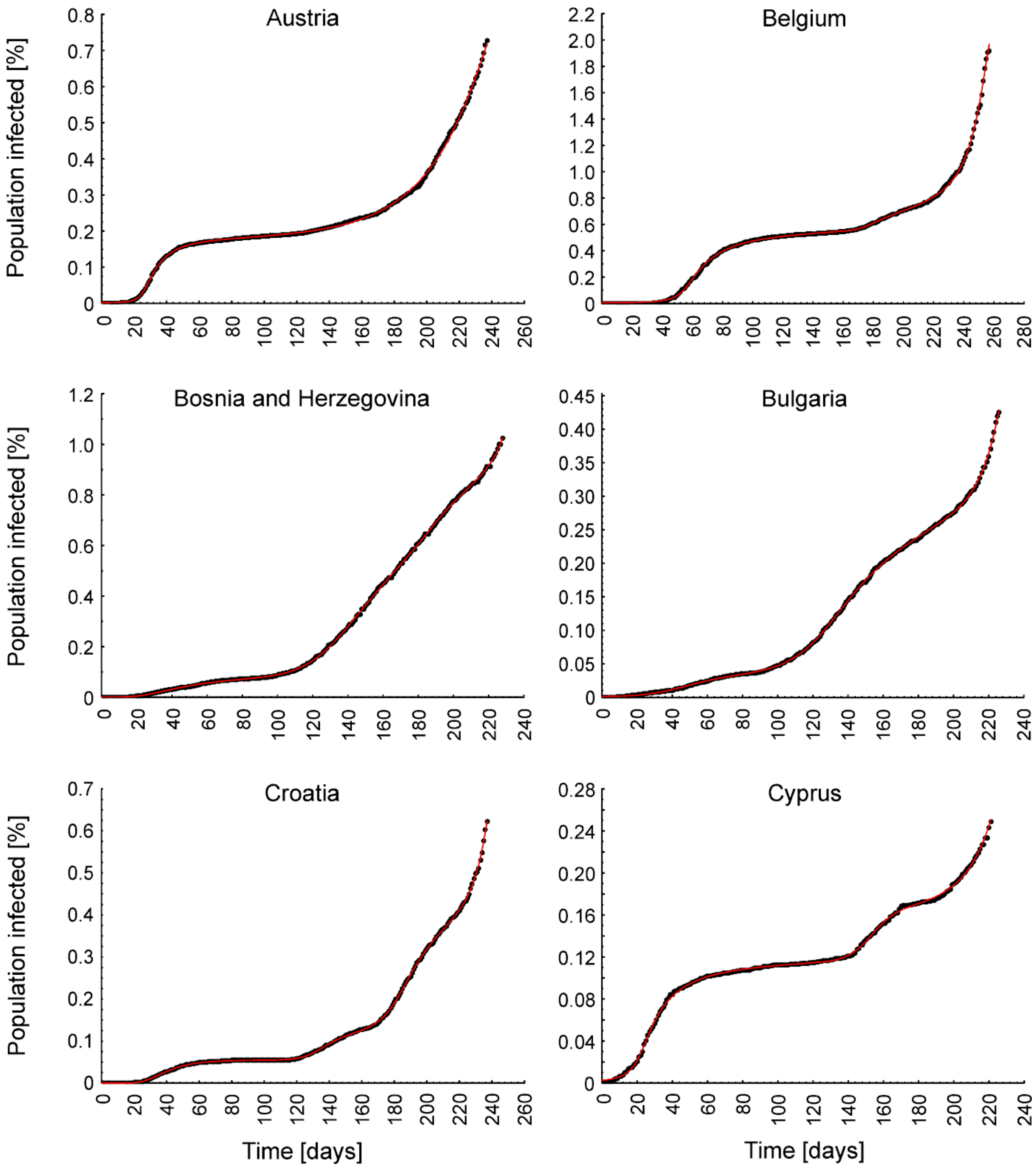

Figure S8. Johnson cumulative density functions (red lines) fitted to the data on COVID-19 trajectories (cumulative epidemic curves) in European countries.

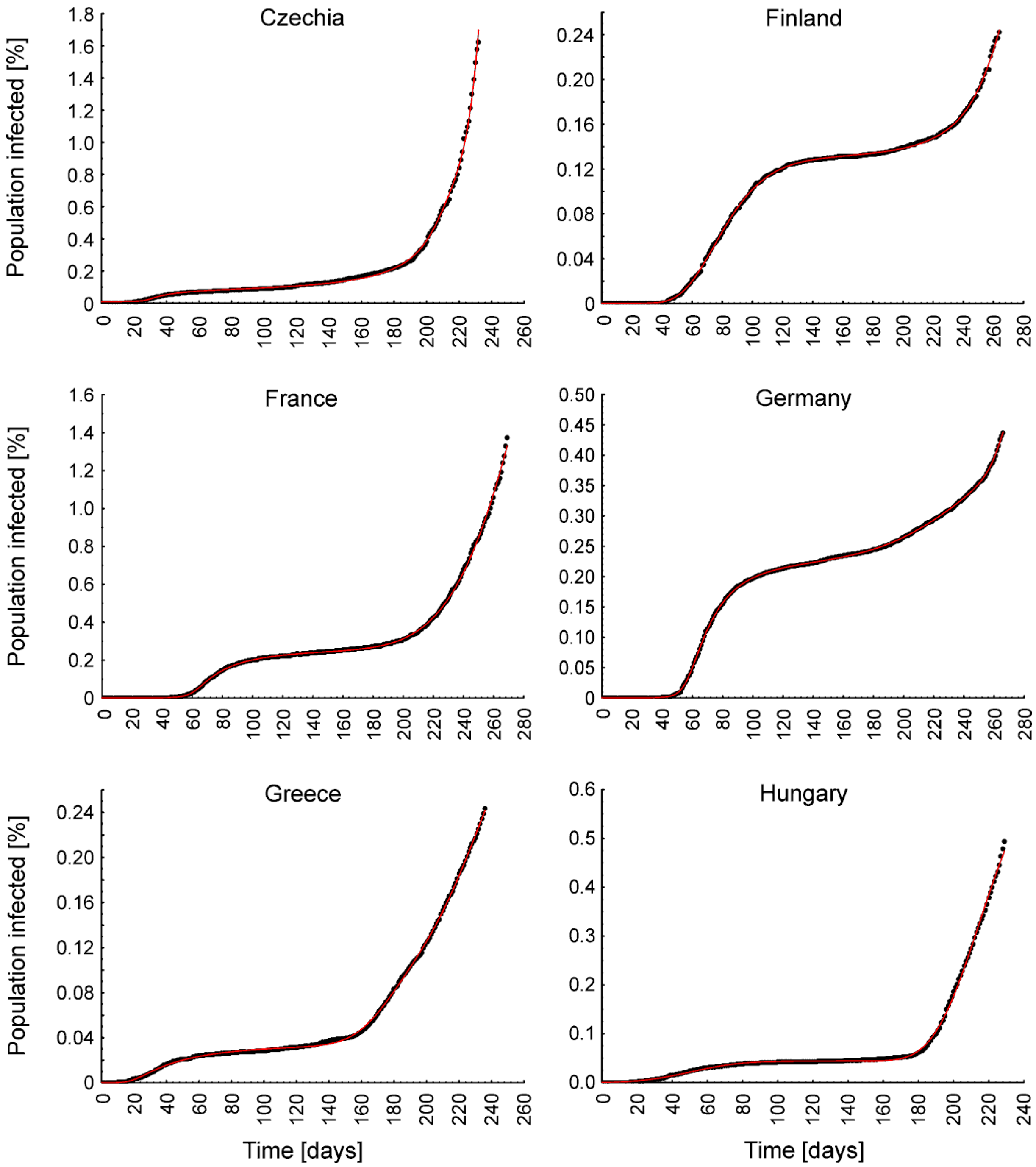

Figure S8 continued. Johnson cumulative density functions (red lines) fitted to the data on COVID-19 trajectories (cumulative epidemic curves) in European countries.

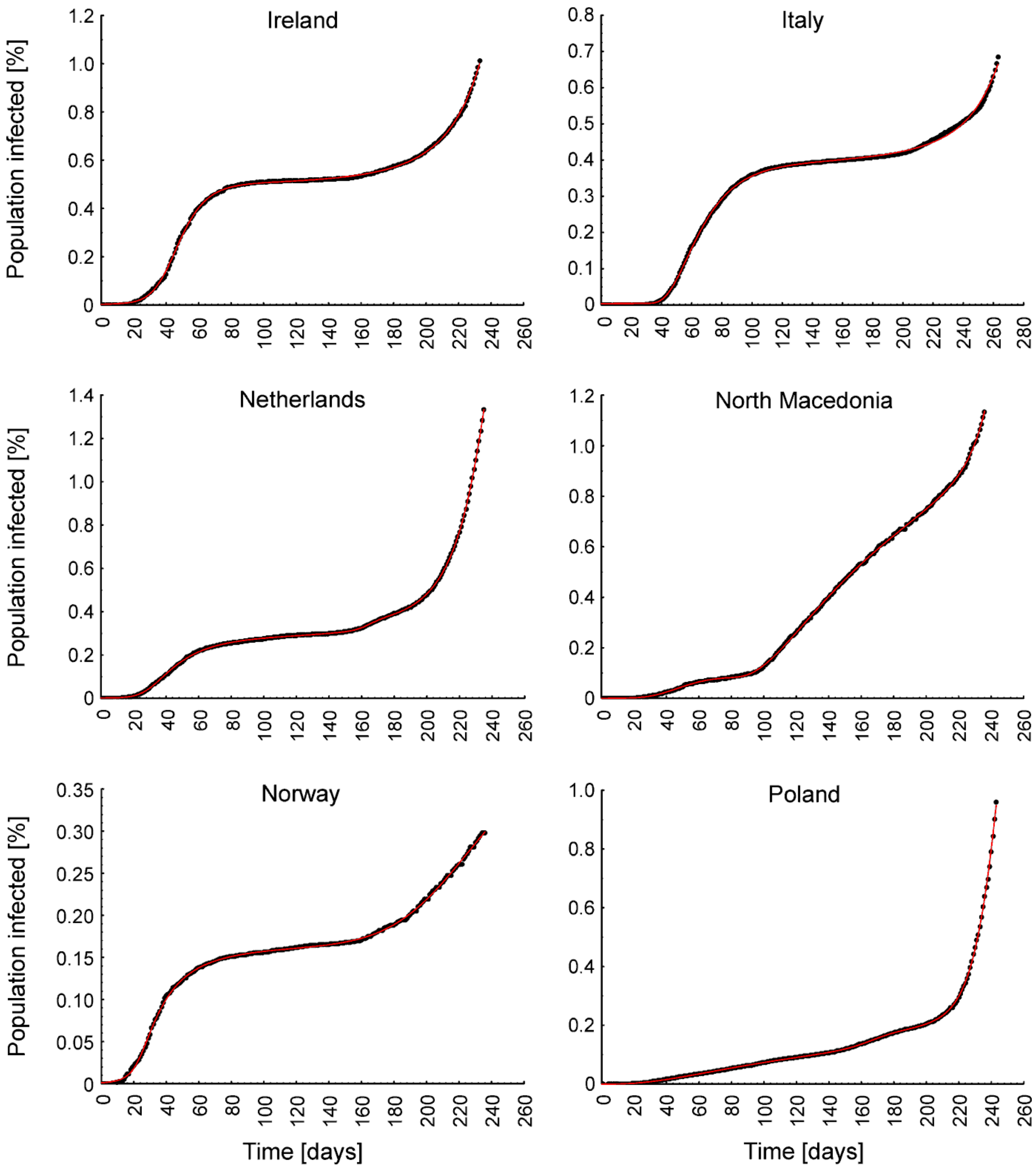

Figure S8 continued. Johnson cumulative density functions (red lines) fitted to the data on COVID-19 trajectories (cumulative epidemic curves) in European countries.

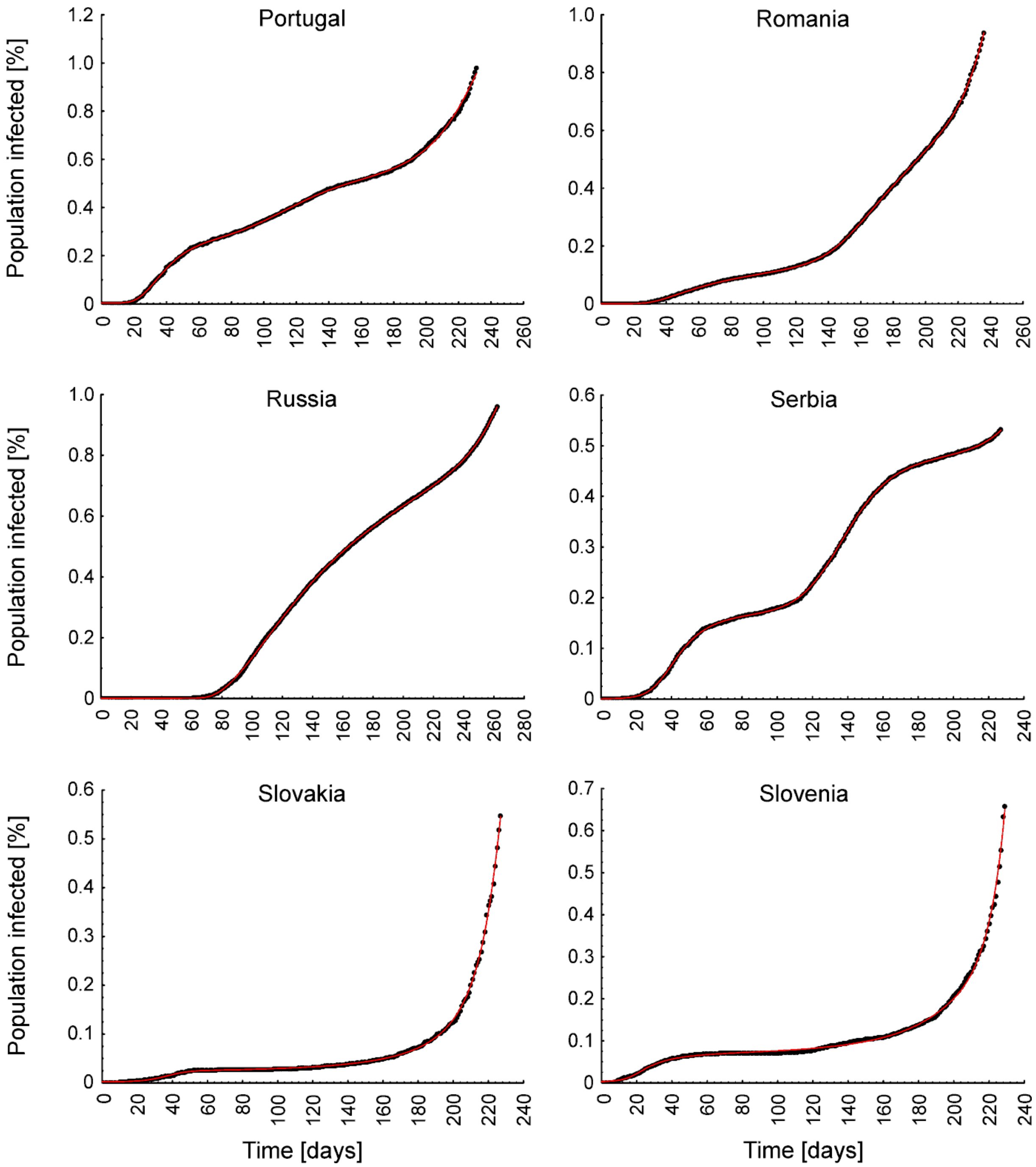

Figure S8 continued. Johnson cumulative density functions (red lines) fitted to the data on COVID-19 trajectories (cumulative epidemic curves) in European countries.

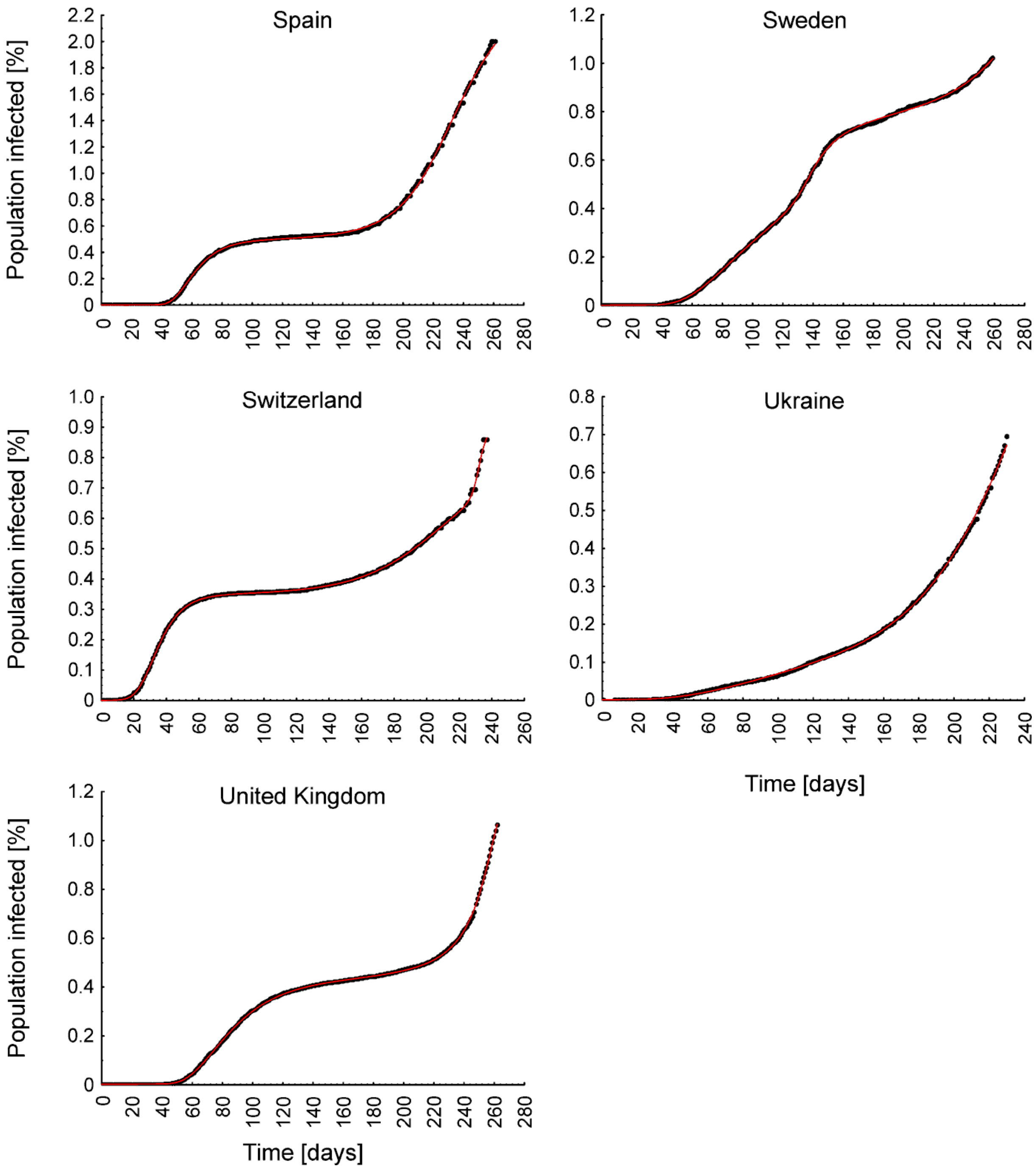

Figure S8 continued. Johnson cumulative density functions (red lines) fitted to the data on COVID-19 trajectories (cumulative epidemic curves) in European countries.

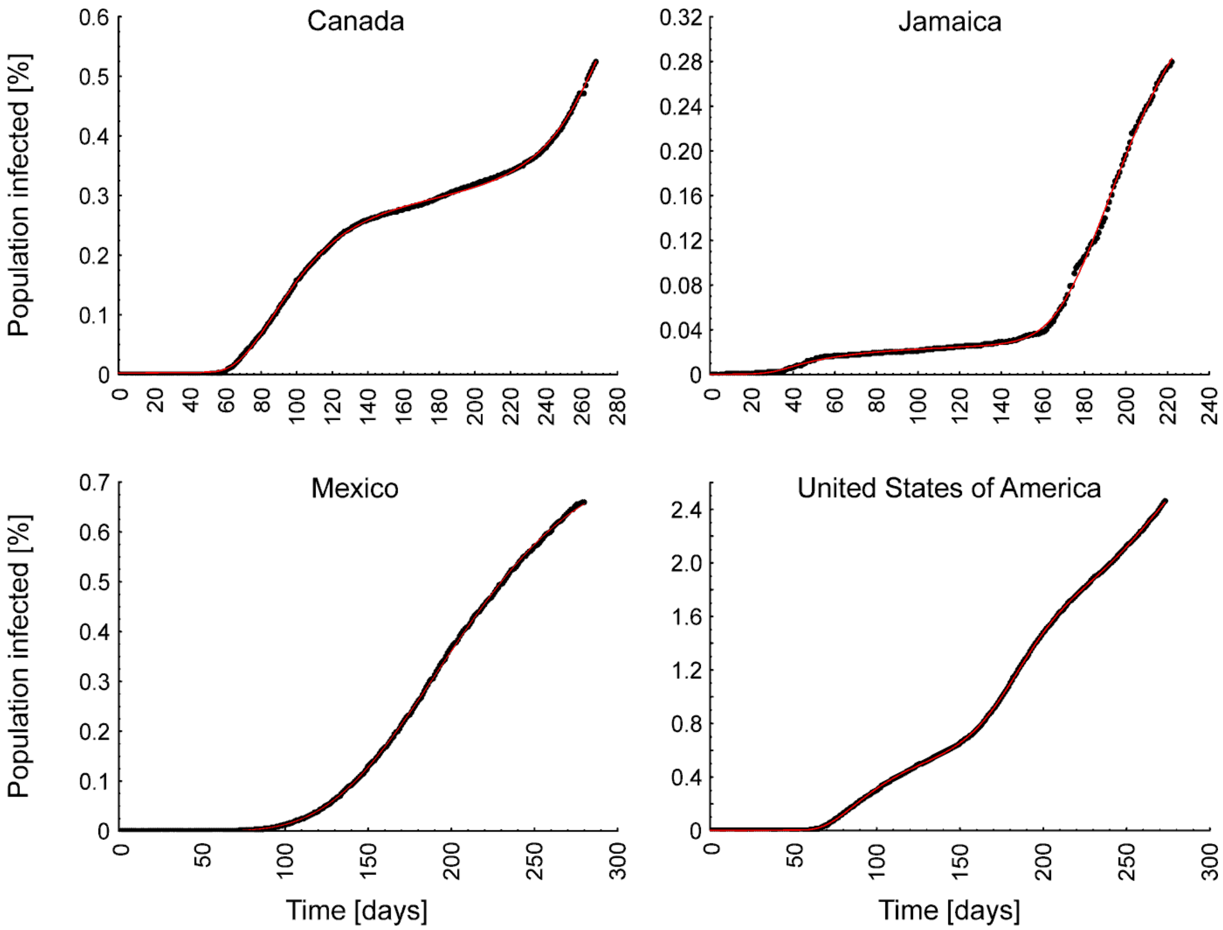

Figure S9. Johnson cumulative density functions (red lines) fitted to the data on COVID-19 trajectories (cumulative epidemic curves) in North American countries.

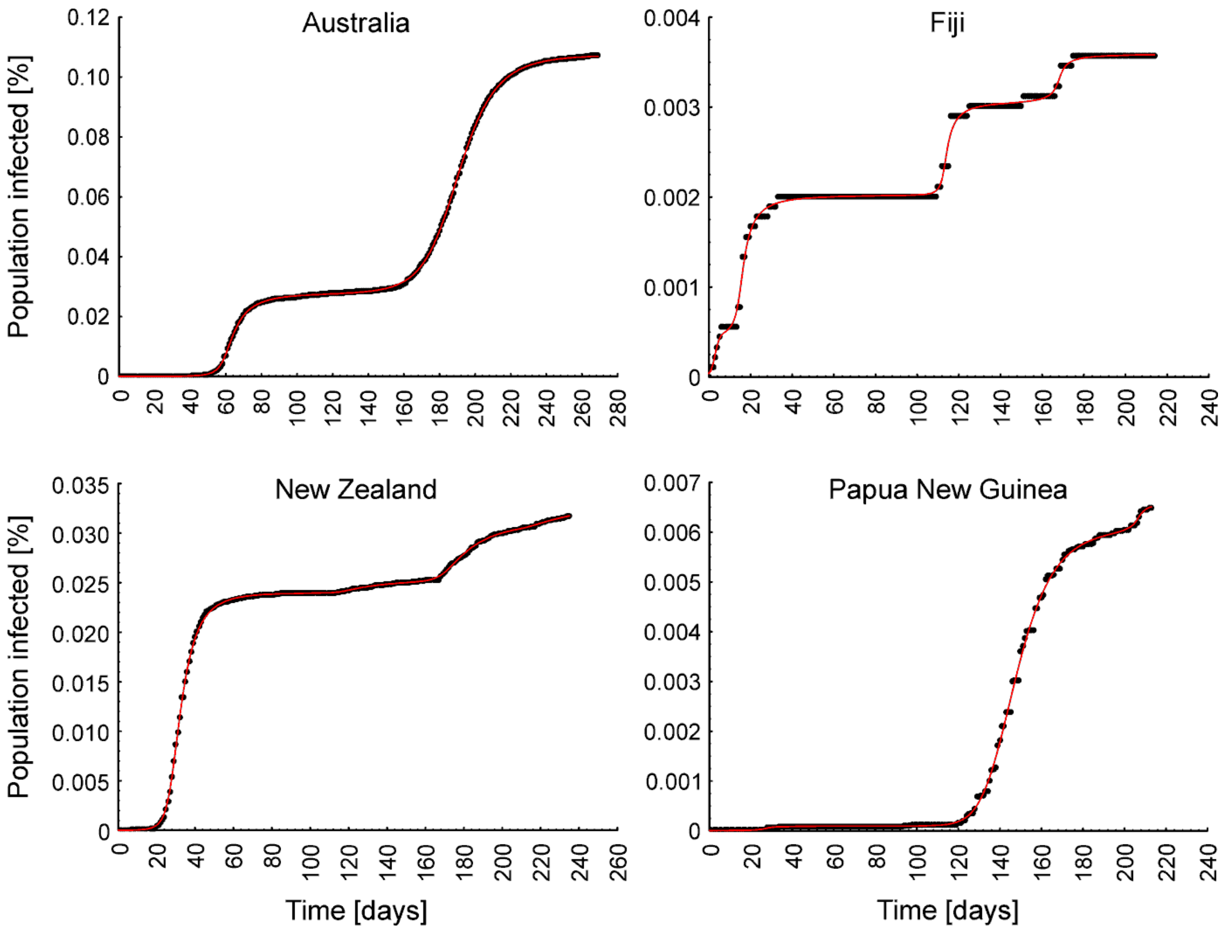

Figure S10. Johnson cumulative density functions (red lines) fitted to the data on COVID-19 trajectories (cumulative epidemic curves) in countries in Oceania.

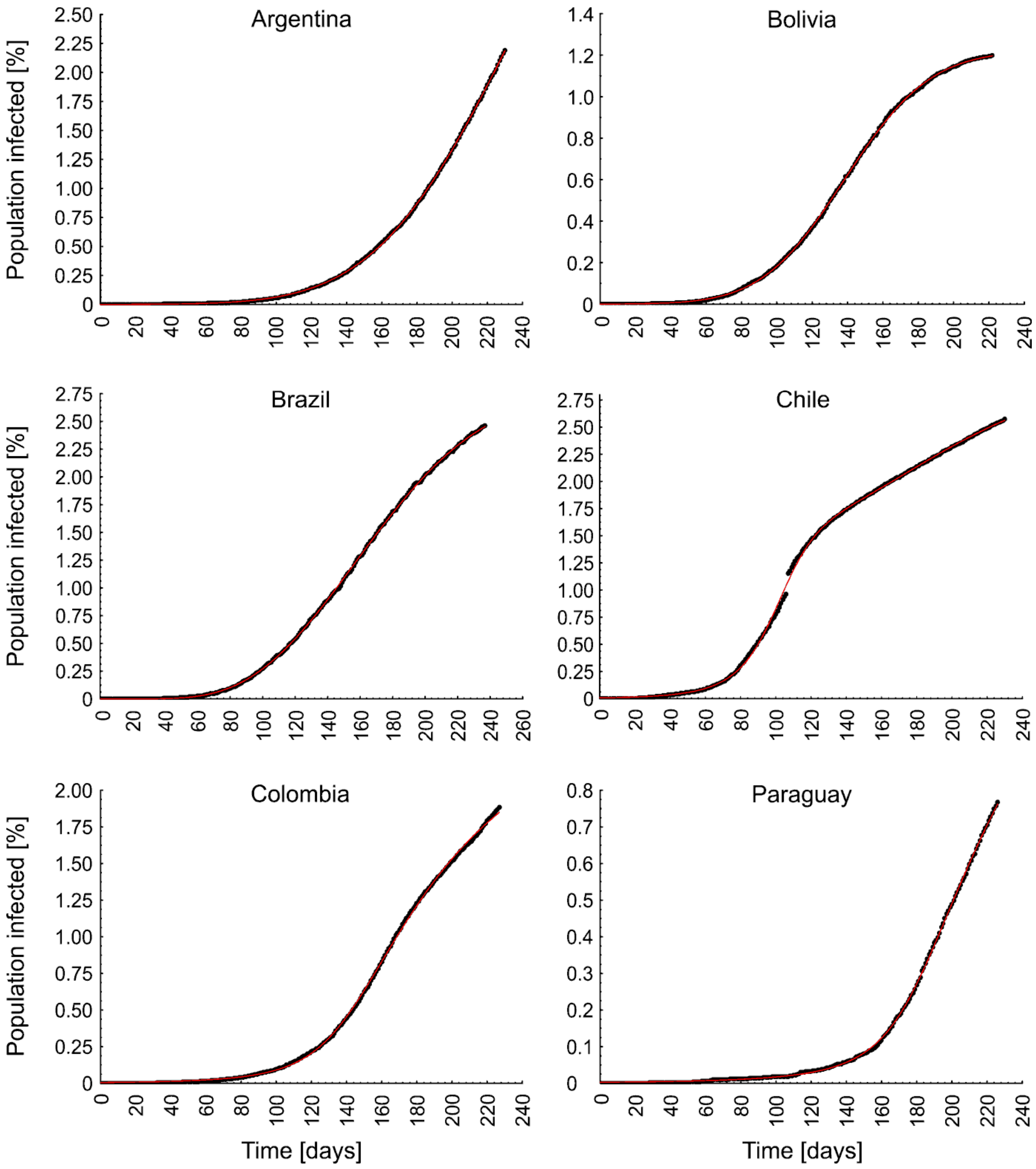

Figure S11. Johnson cumulative density functions (red lines) fitted to the data on COVID-19 trajectories (cumulative epidemic curves) in South American countries.

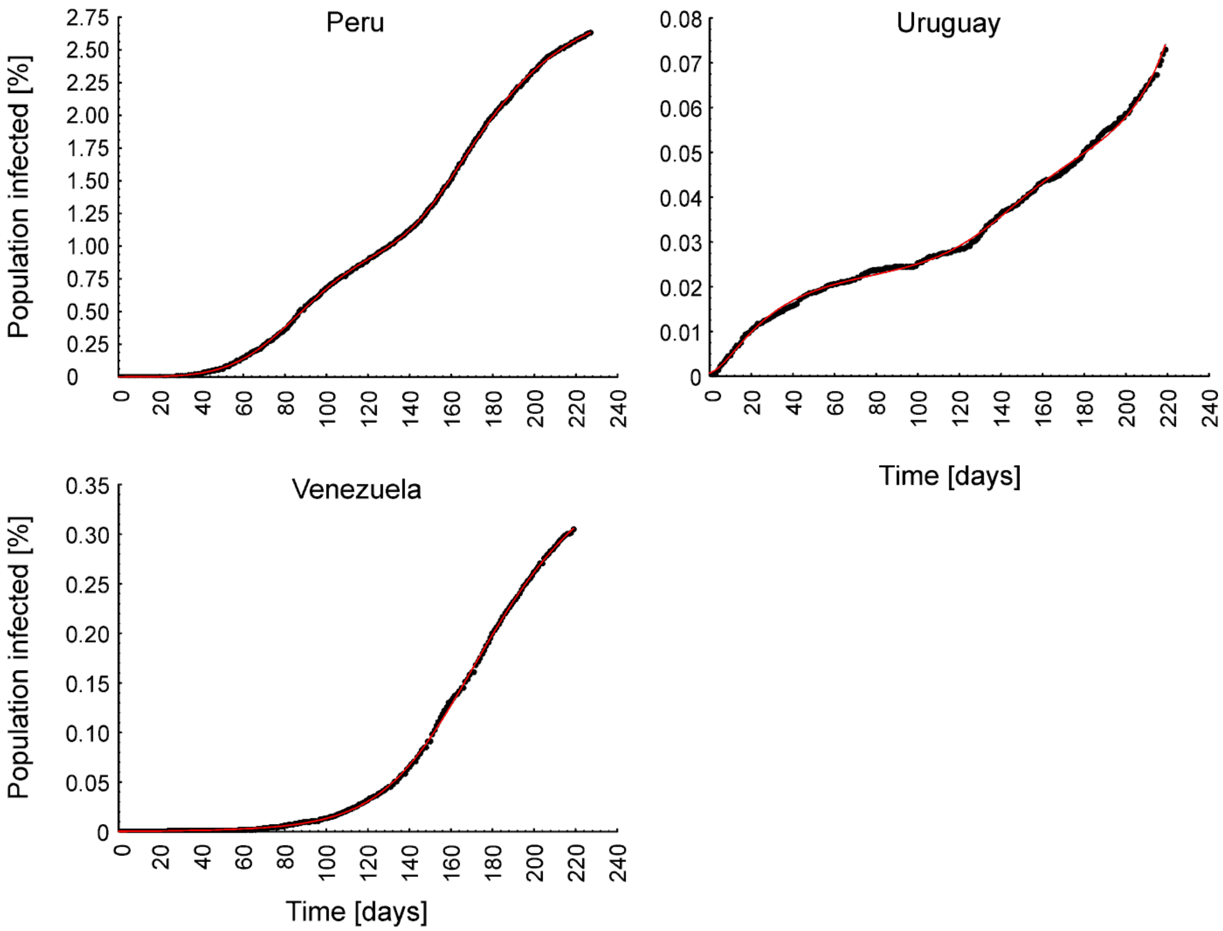

Figure S11 continued. Johnson cumulative density functions (red lines) fitted to the data on COVID-19 trajectories (cumulative epidemic curves) in South American countries.

Table S8. Number of infection waves (N_w) and fitted Johnson cumulative density functions with coefficients of determination (R^2) for each of 80 countries.

Region	Country	N_w	Fitted curve	R^2
Africa	Democratic Republic of Congo	1	$E(t)=0.012907*F_{118.898,70.7757,4.41941,62.2782}(t)$	0.99970
Africa	Egypt	2	$E(t)=0.0972531*F_{125.352,30.6693,0.00050384,3.06174}(t)+0.0122929*F_{260.741,54.8242,1.6964,5.5283}(t)$	0.99992
Africa	Ethiopia	2	$E(t)=0.0724824*F_{164.787,40.9673,0.000152085,22.0159}(t)+0.0382048*F_{298.465,166.884,9.18943,308.199}(t)$	0.99949
Africa	Kenya	2	$E(t)=0.0731944*F_{139.094,39.836,0.00513622,17.513}(t)+0.0474776*F_{233.749,18.9369,0.53122,0.515896}(t)$	0.99970
Africa	Morocco	3	$E(t)=0.0141559*F_{59.9864,17.0538,1.02118,1.91984}(t)+0.0834356*F_{171.999,20.6423,0.00891419,0.000862216}(t)$ $+0.805248*F_{241.998,67.234,1.46249,15.7144}(t)$	0.99970
Africa	Nigeria	1	$E(t)=0.0303193*F_{138.608,46.3461,0.618287,1.23014}(t)$	0.99981
Africa	Somalia	2	$E(t)=0.0205073*F_{71.4445,26.9654,0.669545,0.386906}(t)+0.00498195*F_{202.499,28.8392,1.87492,50.8692}(t)$	0.99936
Africa	South Africa	1	$E(t)=1.17782*F_{139.383,39.416,1.9068,30.4609}(t)$	0.99982
Africa	South Sudan	2	$E(t)=0.0215857*F_{72.4221,51.5002,10.3282,487.988}(t)+0.00710007*F_{187.292,68.6273,0.000721799,0.0014819}(t)$	0.99832
Africa	Sudan	1	$E(t)=0.0340264*F_{115.566,73.978,4.41345,50.7955}(t)$	0.99935
Africa	Zimbabwe	1	$E(t)=0.0619585*F_{159.383,81.6095,10.5104,581.313}(t)$	0.99892
Asia	Afghanistan	1	$E(t)=0.102767*F_{111.081,41.5161,3.53541,51.35}(t)$	0.99980
Asia	Bangladesh	1	$E(t)=0.348623*F_{232.815,234.097,6.96886,154.172}(t)$	0.99987
Asia	Cambodia	2	$E(t)=0.000728299*F_{56.3424,9.91615,5.24715,196.964}(t)+0.000953623*F_{179.33,27.6622,0.900536,120.952}(t)$	0.99873
Asia	China	3	$E(t)=0.00557997*F_{41.168,7.71032,0.00789602,0.335735}(t)+0.000256916*F_{92.3289,16.2606,0.108518,0.011}(t)+$ $+0.000482547*F_{219.72,49.6171,6.06916,438.392}(t)$	0.99922
Asia	India	1	$E(t)=0.722943*F_{230.67,49.6579,0.00668056,0.674791}(t)$	0.99991
Asia	Indonesia	2	$E(t)=0.0762435*F_{153.723,54.2348,0.00615382,0.282283}(t)+0.124304*F_{246.064,63.3083,2.65265,18.7225}(t)$	0.99993
Asia	Iran	3	$E(t)=0.0639611*F_{39.748,10.2852,0.00678327,0.000767145}(t)+0.373225*F_{128.03,49.4976,0.00801498,0.0069}(t)+$ $+0.539529*F_{257.097,48.9543,0.379968,4.75668}(t)$	0.99989

Table S8 continued. Number of infection waves (N_w) and fitted Johnson cumulative density functions with coefficients of determination (R^2) for each of 80 countries.

Region	Country	N_w	Fitted curve	R^2
Asia	Iraq	2	$E(t)=0.131308*F_{129.153,17.7527,0.70030,0.894485}(t)+1.43748*F_{225.835,71.2414,1.4831,6.34081}(t)$	0.99997
Asia	Israel	3	$E(t)=0.178083*F_{47.7947,12.8397,1.23695,2.84856}(t)+1.07353*F_{175.363,53.3451,4.36542,53.2556}(t)+$ $+2.75224*F_{217.455,36.3955,0.00702233,106.2}(t)$	0.99984
Asia	Japan	3	$E(t)=0.0129294*F_{92.9975,17.4181,0.367292,11.0077}(t)+0.0549041*F_{223.087,55.3649,6.65743,198.157}(t)+$ $+0.024011*F_{283.843,31.084,3.54246e-005,2.01303}(t)$	0.99994
Asia	Lebanon	2	$E(t)=0.125332*F_{182.461,11.1118,0.00612455,0.00375655}(t)+1.22259*F_{231.754,51.8948,0.170241,72.7}(t)$	0.99983
Asia	Myanmar	1	$E(t)=0.231405*F_{278.06,158.573,9.57165,353.868}(t)$	0.99929
Asia	Pakistan	2	$E(t)=0.134688*F_{112.052,32.9648,0.292952,8.18107}(t)+0.0498235*F_{263.816,43.7466,0.0064386,0.00248769}(t)$	0.99978
Asia	Philippines	1	$E(t)=0.464499*F_{258.656,148.513,7.92314,264.871}(t)$	0.99954
Asia	Saudi Arabia	1	$E(t)=0.992046*F_{120.187,44.7246,0.754774,2.17753}(t)$	0.99954
Asia	Singapore	2	$E(t)=0.904572*F_{127.87,52.8599,3.45234,28.0657}(t)+0.111026*F_{187.985,8.46479,0.00217685,0.00510362}(t)$	0.99986
Asia	South Korea	2	$E(t)=0.0207541*F_{54.585,36.5873,18.056,1952.21}(t)+0.0355237*F_{233.456,110.451,4.31021,550.873}(t)$	0.99710
Asia	Sri Lanka	2	$E(t)=0.0151098*F_{137.562,47.6004,0.766996,0.792379}(t)+0.0133252*F_{258.358,13.7617,0.555389,62.4503}(t)$	0.99570
Asia	Syria	2	$E(t)=0.0240074*F_{154.728,45.707,0.000259146,89.2068}(t)+0.024922*F_{295.176,230.068,17.5589,1806.64}(t)$	0.99975
Asia	Taiwan	3	$E(t)=0.00190445*F_{68.5568,44.8968,15.0248,9536.08}(t)+0.000229525*F_{242.32,114.578,10.3972,426.548}(t)+$ $+0.00198782*F_{302.701,52.1451,11.127,1434.31}(t)$	0.99931
Asia	Thailand	2	$E(t)=0.00410741*F_{80.1918,11.1578,1.21911,2.76417}(t)+0.00121732*F_{198.408,88.4168,0.00801526,0.327718}(t)$	0.99971
Asia	Turkey	2	$E(t)=0.218575*F_{62.5161,68.5548,9.28242,325.306}(t)+0.44534*F_{236.638,92.6851,0.149186,0.0249466}(t)$	0.99964
Asia	Vietnam	2	$E(t)=0.000353952*F_{89.8007,188.62,141.486,549249}(t)+0.00083114*F_{205.597,92.0535,98.4422,286635}(t)$	0.99917

Table S8 continued. Number of infection waves (N_w) and fitted Johnson cumulative density functions with coefficients of determination (R^2) for each of 80 countries.

Region	Country	N_w	Fitted curve	R^2
Europe	Austria	2	$E(t)=0.17527*F_{37.1483,18.2457,4.85582,72.0865}(t)+1.72405*F_{258.79,56.6247,0.331707,6.14541}(t)$	0.99952
Europe	Belgium	3	$E(t)=0.530032*F_{75.9429,39.6471,7.05439,180.883}(t)+2.30285*F_{254.694,46.6561,0.00106393,1480.24}(t)+$ $+0.130713*F_{194.409,23.6728,2.51733,13.0585}(t)$	0.99947
Europe	Bosnia and Herzegovina	4	$E(t)=0.0739929*F_{53.246,28.8839,2.0354,8.1794}(t)+0.538101*F_{157.025,36.0771,1.3177,4.50388}(t)+$ $+0.309098*F_{199.417,73.828,6.7710,557.55}(t)+1.99763*F_{250.332,38.156,12.058,967.95}(t)$	0.99987
Europe	Bulgaria	3	$E(t)=0.0269585*F_{47.5719,17.7046,0.00146355,0.00307107}(t)+0.249875*F_{156.12,61.25,3.92436,53.962}(t)+$ $+0.372563*F_{227.127,41.3025,0.00119748,1039.35}(t)$	0.99985
Europe	Croatia	4	$E(t)=0.0541405*F_{42.3028,14.5852,1.2054,2.7003}(t)+0.0699166*F_{142.426,19.0722,2.0881,12.541}(t)+$ $+0.265581*F_{195.313,26.1445,2.4423,20.489}(t)+ 1.84424*F_{254.091,25.2158,3.8395,84.862}(t)$	0.99987
Europe	Cyprus	3	$E(t)=0.115092*F_{38.8547,41.5975,11.51,674.693}(t)+0.0427542*F_{153.321,10.3041,0.00302284,0.00496755}(t)+$ $+0.685592*F_{241.113,30.4744,0.166811,83.0336}(t)$	0.99941
Europe	Czechia	3	$E(t)=0.0740236*F_{53.422,57.53,15.7487,1335.66}(t)+0.580889*F_{207.855,84.1427,0.00343905,5776.05}(t)+$ $+3.10353*F_{239.091,46.3418,17.1092,6298.29}(t)$	0.99871
Europe	Finland	2	$E(t)=0.129189*F_{83.5325,23.8704,0.810138,1.19927}(t)+0.343111*F_{277.954,46.5977,1.54627,37.4286}(t)$	0.99976
Europe	France	2	$E(t)=0.261132*F_{90.6015,51.8933,7.89034,221.255}(t)+4.10448*F_{297.808,44.9799,0.00799113,0.0101135}(t)$	0.99946
Europe	Germany	3	$E(t)=0.222966*F_{76.4158,26.2769,3.50858,31.082}(t)+0.181065*F_{233.356,52.564,5.64595e-006,1.93187}(t)+$ $+0.20839*F_{274.96,29.9089,5.70031,159.122}(t)$	0.99993
Europe	Greece	3	$E(t)=0.0348153*F_{61.9783,63.5106,6.17117,112.763}(t)+0.103529*F_{181.986,20.5377,0.00202955,0.00197367}(t)+$ $+0.319133*F_{258.944,42.0616,1.28874,3.10161}(t)$	0.99963
Europe	Hungary	2	$E(t)=0.0441469*F_{50.6497,22.4631,0.0013507,0.00260687}(t)+0.627712*F_{219.048,23.3921,0.37232,0.258861}(t)$	0.99897

Table S8 continued. Number of infection waves (N_w) and fitted Johnson cumulative density functions with coefficients of determination (R^2) for each of 80 countries.

Region	Country	N_w	Fitted curve	R^2
Europe	Ireland	2	$E(t)=0.497913*F_{48.895,15.585,0.699044,2.31044}(t)+2.83079*F_{257.14,40.6004,0.195159,34.0572}(t)$	0.99966
Europe	Italy	2	$E(t)=0.384347*F_{69.7684,22.9268,1.59687,4.86194}(t)+0.731665*F_{270.257,66.8497,0.00872201,404.655}(t)$	0.99940
Europe	Netherlands	3	$E(t)=0.287691*F_{51.6405,28.4184,2.80814,19.3507}(t)+0.0365955*F_{164.368,6.55801,0.00694339,0.00650985}(t)+$ $+3.1847*F_{248.207,36.8726,1.49748,24.4172}(t)$	0.99991
Europe	North Macedonia	3	$E(t)=0.0829176*F_{48.9238,15.1939,0.275836,0.0210793}(t)+1.04243*F_{220.912,154.087,4.53121,51.4201}(t)+$ $+1.51616*F_{247.943,25.1638,2.7862e-005,54.5253}(t)$	0.99988
Europe	Norway	2	$E(t)=0.163703*F_{42.5197,32.1053,5.21729,85.2685}(t)+0.26025*F_{238.661,52.5603,0.848722,2.75276}(t)$	0.99959
Europe	Poland	3	$E(t)=0.132827*F_{102.361,55.4639,0.6934,0.0223507}(t)+0.0668263*F_{166.381,24.9569,0.00170481,11.9708}(t)+$ $+2.34471*F_{250.565,18.5969,0.0915285,2.19396}(t)$	0.99981
Europe	Portugal	3	$E(t)=0.258048*F_{42.5502,17.561,1.63762,5.12868}(t)+0.215328*F_{112.182,32.6685,0.00328498,1.71916}(t)+$ $+1.03118*F_{233.218,58.2843,0.00419217,65.8505}(t)$	0.99961
Europe	Romania	3	$E(t)=0.133803*F_{89.3277,70.5765,4.3139,46.2637}(t)+0.780209*F_{239.423,29.0504,0.0001,42.0172}(t)+$ $+0.783166*F_{268.079,225.04,9.86942,384.941}(t)$	0.99996
Europe	Russia	2	$E(t)=0.824778*F_{175.61,102.354,3.27165,23.6967}(t)+1.02793*F_{281.37,42.8491,1e-005,25.5494}(t)$	0.99995
Europe	Serbia	3	$E(t)=0.170125*F_{47.0028,19.5063,1.85867,9.94486}(t)+0.30192*F_{139.757,23.7944,0.442784,1.54262}(t)+$ $+1.07463*F_{264.903,30.5913,1.51098,28.8457}(t)$	0.99995
Europe	Slovakia	2	$E(t)=0.0210139*F_{36.623,9.83823,0.00649583,0.00114204}(t)+1.44706*F_{237.886,53.2076,8.2407,872.307}(t)$	0.99913
Europe	Slovenia	3	$E(t)=0.0667705*F_{31.0458,22.1511,3.82183,44.5024}(t)+0.0372809*F_{153.216,45.378,3.13201,21.426}(t)+$ $+3.10237*F_{240.203,26.5982,0.00343161,356.051}(t)$	0.99779
Europe	Spain	2	$E(t)=0.488605*F_{64.7353,15.3781,1.33086,3.32056}(t)+2.13144*F_{247.923,61.8614,2.724,31.7884}(t)$	0.99955

Table S8 continued. Number of infection waves (N_w) and fitted Johnson cumulative density functions with coefficients of determination (R^2) for each of 80 countries.

Region	Country	N_w	Fitted curve	R^2
Europe	Sweden	3	$E(t)=0.489846*F_{108.244,45.1005,1.56794,4.67754}(t)+0.297079*F_{141.705,25.0865,2.42091,46.5798}(t)+$ $+0.814077*F_{279.847,54.6232,0.00622152,10.7975}(t)$	0.99987
Europe	Switzerland	3	$E(t)=0.347761*F_{36.8963,13.1397,1.09462,2.6412}(t)+0.45431*F_{209.667,50.124,0.0052421,3.03597}(t)+$ $+0.20404*F_{232.143,4.56782,0.00999514,3.2024}(t)$	0.99971
Europe	Ukraine	2	$E(t)=0.0974848*F_{101.361,42.2545,0.591188,0.0564594}(t)+2.53384*F_{293.439,91.9928,1.15615,4.51605}(t)$	0.99958
Europe	United Kingdom	2	$E(t)=0.424517*F_{91.7018,30.9686,1.67723,5.51722}(t)+1.11024*F_{258.58,45.4118,3.7409e-005,286.879}(t)$	0.99986
North America	Canada	2	$E(t)=0.280839*F_{104.241,32.1361,1.5151,4.35129}(t)+0.517431*F_{270.257,68.205,0.001574,94.8645}(t)$	0.99971
North America	Jamaica	2	$E(t)=0.0293465*F_{89.9085,126.443,14.6287,1088.16}(t)+0.39252*F_{217.746,51.5602,2.46008,14.1724}(t)$	0.99913
North America	Mexico	1	$E(t)=0.744036*F_{206.989,59.3047,0.429441,0.0393411}(t)$	0.99987
North America	United States of America	3	$E(t)=0.550603*F_{103.596,30.6493,1.66289,5.29818}(t)+1.19572*F_{185.159,30.4121,0.156334,1.74146}(t)+$ $+1.84218*F_{312.212,113.282,4.70041,76.1051}(t)$	0.99997
Oceania	Australia	2	$E(t)=0.027695*F_{67.0193,15.1412,4.91014,91.1291}(t)+0.0797208*F_{191.801,22.0842,0.650094,5.48317}(t)$	0.99996
Oceania	Fiji	3	$E(t)=0.00202481*F_{14.5684,13.953,0.000356559,76.545}(t)+0.00101567*F_{115.978,7.33466,6.0608,148.182}(t)+$ $+0.000546777*F_{168.152,20.8587,0.000183706,19851.4}(t)$	0.99497
Oceania	New Zealand	4	$E(t)=0.0239738*F_{34.354,10.3045,2.70678,22.131}(t)+0.00130462*F_{141.203,39.8374,6.6160,139.061}(t)+$ $+0.00603697*F_{189.027,29.2594,4.9602,72.4903}(t)+0.00156923*F_{244.737,35.1765,3.403,25.9784}(t)$	0.99982
Oceania	Papua New Guinea	3	$E(t)=9.57574e-05*F_{27.1931,90.834,0.005145,1.1830e+007}(t)+0.005913*F_{149.554,16.4096,0.9896,2.9169}(t)+$ $+ 0.000620503*F_{206.608,33.8333,0.0014881,108048}(t)$	0.99949

Table S8 continued. Number of infection waves (N_w) and fitted Johnson cumulative density functions with coefficients of determination (R^2) for each of 80 countries.

Region	Country	N_w	Fitted curve	R^2
South America	Argentina	2	$E(t)=0.17527*F_{37.1483,18.2457,4.85582,72.0865}(t)+1.72405*F_{258.79,56.6247,0.331707,6.14541}(t)$	0.99992
South America	Bolivia	1	$E(t)=1.22183*F_{139.642,39.0547,0.0962302,0.317568}(t)$	0.99992
South America	Brazil	1	$E(t)=2.74199*F_{167.255,53.0097,0.284768,0.236245}(t)$	0.99992
South America	Chile	2	$E(t)=1.81944*F_{102.247,28.2296,0.0048419,8.30444}(t)+1.30239*F_{236.372,83.3318,1.9275,7.26942}(t)$	0.99966
South America	Colombia	1	$E(t)=2.79391*F_{232.005,160.021,6.71011,152.059}(t)$	0.99971
South America	Paraguay	1	$E(t)=1.92033*F_{351.719,398.677,15.815,1351.45}(t)$	0.99979
South America	Peru	2	$E(t)=0.933518*F_{86.145,25.0466,0.00462452,0.00837006}(t)+2.06124*F_{190.799,55.982,2.64435,17.9234}(t)$	0.99995
South America	Uruguay	3	$E(t)=0.0240813*F_{37.6414,38.1737,3.22348,22.897}(t)+0.0214958*F_{146.316,28.0122,0.144591,0.00416435}(t)+$ $+0.109209*F_{238.163,66.7847,3.37775,455.887}(t)$	0.99855
South America	Venezuela	1	$E(t)=0.398944*F_{188.667,61.1926,1.55804,8.43544}(t)$	0.99978

Table S9. Basic parameters of the first infection wave dynamics calculated using Johnson cumulative density functions fitted to the pandemic wave in a given country. *S* - skewness parameter of the fitted Johnson CDF, P_{inf}- percentage of infections during the epidemic wave,$Q_{2.5\%}$ - the day the infection wave started,$Q_{50\%}$ - the day that half the total percentage of infections during a given wave was reached,$Q_{97.5\%}$- the day the infection wave ended, M - the day the peak occurred, t_i - the duration of the wave increase, t_d - the duration of the wave decrease, *T* - the wave duration, and *A*- the asymmetry of the infection wave.

Region	Country	S	P_{inf}	$Q_{2.5\%}$	$Q_{50\%}$	$Q_{97.5\%}$	M	t_i	t_d	T	A
Africa	Democratic Republic of Congo	4.42	0.01291	39	103	293	90	51	203	255	3.96
Africa	Egypt	0.001	0.09725	63	125	187	125	62	62	124	1.01
Africa	Ethiopia	0.0002	0.07248	84	165	246	165	81	81	162	0.99
Africa	Kenya	0.01	0.07319	60	139	218	139	79	79	158	1.00
Africa	Morocco	1.02	0.01416	34	57	100	53	19	47	66	2.55
Africa	Nigeria	0.62	0.03032	59	135	242	128	69	114	183	1.65
Africa	Somalia	0.67	0.02051	29	68	133	60	31	73	104	2.34
Africa	South Africa	1.91	1.17782	74	136	227	133	59	94	153	1.58
Africa	South Sudan	10.33	0.02159	30	61	188	54	24	134	158	5.58
Africa	Sudan	4.41	0.03403	48	95	306	74	26	232	258	8.90
Africa	Zimbabwe	10.51	0.06196	82	143	337	134	52	203	255	3.94
Asia	Afghanistan	3.54	0.10277	54	104	209	100	46	109	155	2.38
Asia	Bangladesh	6.97	0.34862	65	166	811	110	45	701	746	15.57
Asia	Cambodia	5.25	0.00073	43	55	78	52	9	26	35	2.76
Asia	China	0.01	0.00558	26	41	56	42	16	14	31	0.90
Asia	India	0.01	0.72294	132	231	330	231	99	99	198	0.99
Asia	Indonesia	0.01	0.07624	47	154	261	154	107	107	214	0.99
Asia	Iran	0.01	0.06396	20	40	60	40	20	20	40	0.98
Asia	Iraq	0.70	0.13131	100	127	169	123	23	46	69	2.03
Asia	Israel	1.24	0.17808	30	46	79	44	14	35	49	2.42
Asia	Japan	0.37	0.01293	59	93	129	92	33	37	70	1.14
Asia	Lebanon	0.01	0.12533	161	182	204	182	21	22	44	1.04
Asia	Myanmar	9.57	0.23141	175	234	650	204	29	446	474	15.56
Asia	Pakistan	0.29	0.13469	48	111	181	111	63	70	133	1.10
Asia	Philippines	7.92	0.46450	115	226	602	206	91	396	487	4.33
Asia	Saudi Arabia	0.75	0.99205	43	116	221	110	67	111	178	1.67
Asia	Singapore	3.45	0.90457	75	114	265	97	22	168	190	7.82
Asia	South Korea	18.06	0.02075	35	46	130	41	6	89	95	14.08
Asia	Sri Lanka	0.77	0.01511	63	131	247	118	55	129	184	2.35
Asia	Syria	0.00	0.02401	70	155	240	155	85	85	170	0.99
Asia	Taiwan	15.02	0.00190	18	66	135	65	47	70	117	1.48
Asia	Thailand	1.22	0.00411	64	78	107	75	11	32	43	3.01
Asia	Turkey	9.28	0.21858	18	43	224	28	10	196	206	19.95
Asia	Vietnam	141.49	0.00035	22	65	304	60	38	244	282	6.46

Table S9 continued. Basic parameters of the first infection wave dynamics calculated using the Johnson cumulative density function fitted to the pandemic wave in a given country. S - skewness parameter of the fitted Johnson CDF, P_{inf} - percentage of infections during an epidemic wave $Q_{2.5\%}$ - the day the infection wave started, $Q_{50\%}$ - the day that half the total percentage of infections during a given wave was reached, $Q_{97.5\%}$ - the day the infection wave ended, M - the day the peak occurred, t_i - the duration of the wave increase, t_d - the duration of the wave decrease, T - the wave duration, and A - the asymmetry of the infection wave.

Region	Country	S	P_{inf}	$Q_{2.5\%}$	$Q_{50\%}$	$Q_{97.5\%}$	M	t_i	t_d	T	A
Europe	Austria	4.86	0.17527	18	33	83	29	11	54	64	5.01
Europe	Belgium	7.05	0.53003	40	66	171	60	20	111	131	5.47
Europe	Bosnia and Herzegovina	2.04	0.07399	19	46	128	35	16	93	108	5.82
Europe	Bulgaria	0.00	0.02696	13	48	82	49	36	33	69	0.92
Europe	Croatia	1.21	0.05414	21	40	78	35	14	43	56	3.14
Europe	Cyprus	11.51	0.11509	4	30	130	25	21	105	126	4.94
Europe	Czechia	15.75	0.07402	22	39	175	31	9	144	153	16.33
Europe	Finland	0.81	0.12919	46	81	139	75	29	64	93	2.17
Europe	France	7.89	0.26113	51	77	215	67	16	148	164	9.29
Europe	Germany	3.51	0.22297	48	70	144	63	15	81	95	5.55
Europe	Greece	6.17	0.03482	15	44	221	27	12	194	207	15.67
Europe	Hungary	0.00	0.04415	7	51	95	51	44	44	88	0.98
Europe	Ireland	0.70	0.49791	22	48	84	46	24	38	62	1.54
Europe	Italy	1.60	0.38435	40	65	127	57	17	70	87	4.13
Europe	Netherlands	2.81	0.28769	18	45	124	37	19	87	106	4.60
Europe	North Macedonia	0.28	0.08292	21	48	81	47	26	34	59	1.31
Europe	Norway	5.22	0.16370	10	35	122	28	18	94	112	5.33
Europe	Poland	0.69	0.13283	21	93	229	64	43	165	208	3.88
Europe	Portugal	1.64	0.25805	20	39	87	33	13	54	67	4.12
Europe	Romania	4.31	0.13380	29	69	273	46	17	227	244	13.17
Europe	Russia	3.27	0.82478	78	147	445	109	31	336	366	10.96
Europe	Serbia	1.86	0.17013	19	44	95	40	21	55	76	2.56
Europe	Slovakia	0.01	0.02101	17	37	56	37	20	19	39	0.96
Europe	Slovenia	3.82	0.06677	5	26	86	20	15	66	81	4.36
Europe	Spain	1.33	0.48861	43	62	103	57	14	46	59	3.37
Europe	Sweden	1.57	0.48985	49	99	221	83	34	138	172	4.09
Europe	Switzerland	1.09	0.34776	17	35	68	32	15	36	51	2.39
Europe	Ukraine	0.59	0.09748	35	96	196	83	48	113	161	2.33
Europe	United Kingdom	1.68	0.42452	52	85	170	74	22	96	118	4.30
North America	Canada	1.52	0.28084	62	98	184	87	25	97	123	3.84
North America	Jamaica	14.63	0.02347	21	57	363	36	15	327	341	22.39
North America	Mexico	0.43	0.74404	105	202	335	191	86	144	229	1.68
North America	United States of America	1.66	0.55060	64	97	181	86	22	95	117	4.39

Table S9 continued. Basic parameters of the first infection wave dynamics calculated using the Johnson cumulative density function fitted to the pandemic wave in a given country. *S* - skewness parameter of the fitted Johnson CDF, P_{inf} - percentage of infections during an epidemic wave $Q_{2.5\%}$ - the day the infection wave started, $Q_{50\%}$ - the day that half the total percentage of infections during a given wave was reached, $Q_{97.5\%}$ - the day the infection wave ended, M - the day the peak occurred, t_i - the duration of the wave increase, t_d - the duration of the wave decrease, *T* - the wave duration, and *A* - the asymmetry of the infection wave.

Region	Country	S	P_{inf}	$Q_{2.5\%}$	$Q_{50\%}$	$Q_{97.5\%}$	M	t_i	t_d	T	A
Oceania	Australia	4.91	0.02770	49	64	103	62	13	41	54	3.16
Oceania	Fiji	0.00	0.00202	-13	15	42	15	28	27	55	0.97
Oceania	New Zealand	2.71	0.02397	21	32	60	30	9	30	39	3.15
Oceania	Papua New Guinea	0.01	0.00010	-25	27	79	27	52	52	104	1.01
South America	Argentina	0.00	4.53221	114	233	351	233	119	118	237	0.99
South America	Bolivia	0.10	1.22183	64	139	219	138	74	81	154	1.09
South America	Brazil	0.28	2.74199	70	165	279	157	87	122	208	1.40
South America	Chile	0.00	1.81944	45	102	159	100	55	59	114	1.08
South America	Colombia	6.71	2.79391	93	191	622	160	67	462	528	6.90
South America	Paraguay	15.82	1.92033	135	250	1196	197	62	999	1061	16.10
South America	Peru	0.00	0.93352	37	86	135	86	49	49	98	1.01
South America	Uruguay	3.22	0.02408	1	27	138	13	12	125	137	10.52
South America	Venezuela	1.56	0.39894	93	180	334	170	77	164	241	2.13

Fitting Johnson curves to the ongoing wave: forecasting possibilities

The accuracy of forecasts is discussed on the basis of data relating to the first wave of infections in the United Kingdom. The UK was selected because it is both highly populated (67,886,004) and, among European countries, has carried out the most test, with a mean of 126.33 tests per 1,000 since the beginning of the pandemic up to 19 October 2020.

The first wave of infections was described using the Johnson Cumulative Density Curve, and parameters P_{inf}, $Q_{2.5\%}$, M, $Q_{97.5\%}$, T_i, T_d and T were calculated. The values obtained for $Q_{2.5\%}$, M and $Q_{97.5\%}$ indicated that the first wave of infections in the UK started on the 51st and finished on the 170th day of the epidemic, while the wave peaked on the 74th day. Then, a series of forecasts were made by fitting Johnson distribution curves to the data cut to the first 60, 70, 80, 90, 100, 110, 120, 130, 140, 150 and 160 days of the epidemic. P_{inf}, $Q_{2.5\%}$, M, $Q_{97.5\%}$, T_i, T_d and T were calculated for each forecast; the results are listed in Table S10. In addition, the percentage difference between the actual and predicted percentage of infections on each day was calculated for each forecast (starting from the forecast day to its end at t=170; see Fig. S12). Fig. S13 illustrates the example forecasts based on days 60, 90, 210 and 150 compared to the Johnson distribution curve fitted to the complete data.

The results showed that the predicted parameters describing the day of the wave peak, the day the infection wave ended, and the duration of the wave, its increase and decrease did not differ much from the values obtained using the complete dataset for the first wave of infections in the UK (Table S10). However, the percentages of the population infected predicted using only days 70 and 80 were approximately 0.1% lower than the actual figures. This means that, if recalculated to the number of infections, the predicted number of infections was approximately 68,000 lower than the actual number. This suggests that such early predictions (prior to the peak) made using Johnson CDF fitting should focus on predicting the day of the peak rather than the number of infections. Additionally, for early forecasts, the predicted daily percentages of infections differed from the actual percentages of infections by more than 10% in the longer term (Fig. S12). However, the predictions made after the wave had peaked were consistent with the observations.

It also needs to be highlighted that this curve fitting method was designed primarily not for making forecasts but rather for obtaining easily interpretable parameters describing the past trajectory of COVID-19 infections. Thus, extreme caution is advisable when forecasting the future trajectory of the infection wave and its parameters (see the Discussion).

Table S10. Parameters describing the percentage of the infected population (P_{inf}), the day the infection wave started ($Q_{2.5\%}$), the day the wave peaked (M), the day the infection wave ended ($Q_{97.5\%}$), the duration of the wave increase (T_i), the duration of the wave decrease (T_d) and the duration of the wave of infections (T) for each forecast using a different number of days from the beginning of the epidemic (t=0) to the end of the first infection wave (t=170).

Days used	P_{inf}	$Q_{2.5\%}$	M	$Q_{97.5\%}$	T_i	T_d	T
60 (9 days after wave start)	0.434241	51	74	157	23	83	106
70	0.334370	49	69	165	20	96	116
80	0.338205	49	69	169	20	100	120
90	0.414673	50	75	161	25	86	111
100	0.444636	51	75	170	24	95	119
110	0.459856	51	75	180	24	105	129
120	0.444350	51	75	170	24	95	120
130	0.433287	50	75	163	25	88	112
140	0.430665	50	76	161	26	85	111
150	0.433254	50	75	163	25	88	113
160	0.437829	50	75	168	25	93	118
170 (complete wave)	0.424517	52	74	170	22	96	118

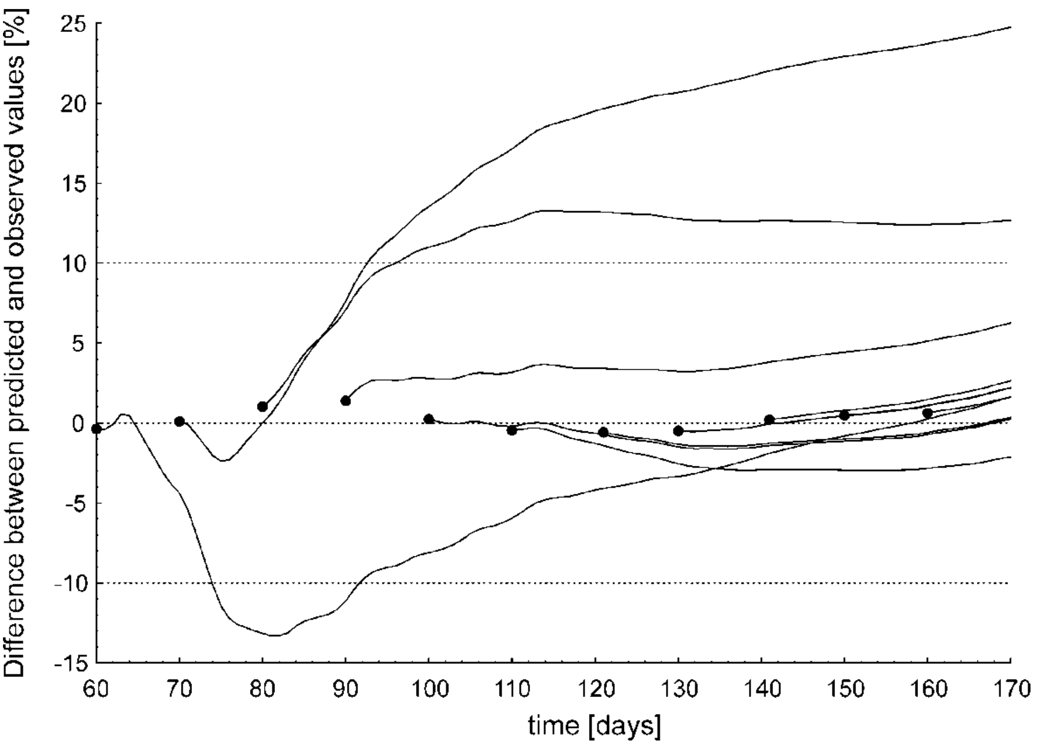

Figure S12. The percentage difference between the actual percentage of the population infected and the predicted percentage of the population infected for 11 forecasts using different numbers of days (60, 70, 80, 90, 100, 110, 120, 130, 140, 150 and 160), starting from the forecast day to the end at t=170.

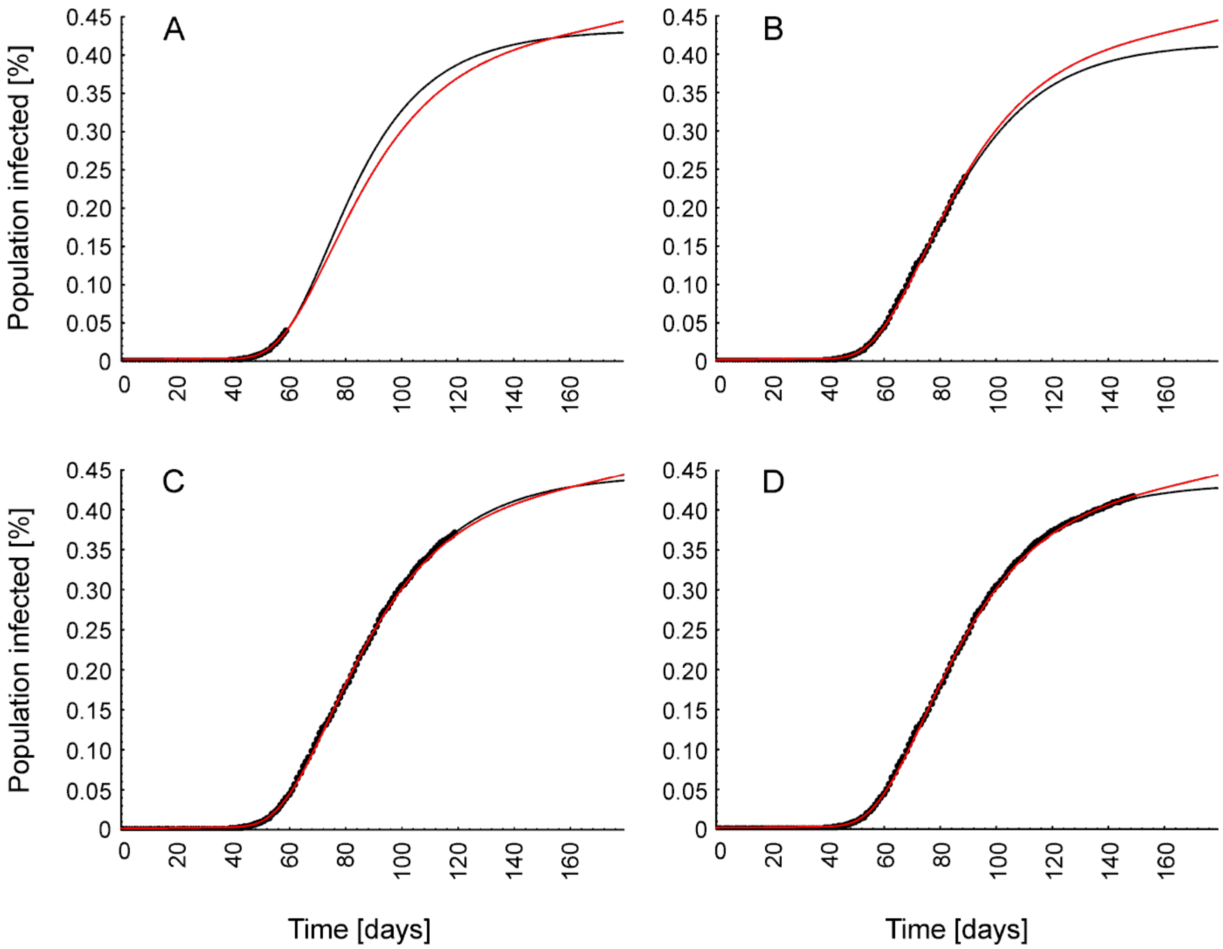

Figure S13. Examples of predictions of the future trajectory of the infection wave (black line) using data (black dots) from days 60 (A), 90 (B), 120 (C) and 150 (D) in comparison to the actual trajectory of the infection wave (red line).
